# Circulation and Codetections of Influenza Virus, SARS-CoV-2, Respiratory Syncytial Virus, Rhinovirus, Adenovirus, Bocavirus, and Other Respiratory Viruses During 2022–2023 Season in Latvia

**DOI:** 10.3390/v16111650

**Published:** 2024-10-22

**Authors:** Inara Kampenusa, Baiba Niedre-Otomere, Julija Trofimova, Ilva Pole, Gatis Pakarna, Oksana Savicka, Sergejs Nikisins

**Affiliations:** 1 National Microbiology Reference Laboratory of Latvia, Laboratory “Latvian Centre of Infectious Diseases” Laboratory Service, Riga East University Hospital, Linezera Street 3, LV-1006 Riga, Latvia; inara.kampenusa@aslimnica.lv (I.K.);; 2 Department of Infectology, Riga Stradins University, Riga East University Hospital, Linezera Street 3, LV-1006 Riga, Latvia

**Keywords:** respiratory viruses, bocavirus, enterovirus, influenza viruses, SARS-CoV-2, respiratory syncytial virus, rhinovirus, adenovirus, patients’ median age

## Abstract

This retrospective study analysed the routine data obtained by multiplex real-time RT-qPCR methods for respiratory virus detection. A total of 4814 respiratory specimens collected during 1 September 2022–31 August 2023 were included in the study. A total of 38% of the specimens were positive for at least one target, with the incidence maximum (82%) for the small children (age group 0–4 years). The five dominant virus groups were rhinovirus (RV, 12%), influenza virus A (IAV, 7%), adenovirus (AdV, 6%), respiratory syncytial virus (RSV, 5%), and severe acute respiratory syndrome coronavirus 2 (SARS-CoV-2, 5%). The specimens with multi-detections represented 19% of the positives, unevenly distributed (n = 225, 56, 43, 24) among the age groups 0–4, 5–14, 15–64, and 65< years, respectively. The dominant virus groups in multi-positive specimens were RV (53%), AdV (43%), and bocavirus (BoV, 35%)—in mutual pairs as well as all three together—followed by RSV (21%), and IAV (15%). Our study focused on the specimens with codetections and provides an insight into the variety of the respiratory virus interactions in Latvia during the first year since pandemic-related social restriction measures were eased. The observations also emphasise the need to consider the differentiation between rhinoviruses and enteroviruses, especially for the youngest patients in the age group 0–4.

## 1. Introduction

For a few years, the severe acute respiratory syndrome coronavirus 2 (SARS-CoV-2) outcompeted the influenza viruses as the main targets of the respiratory virus monitoring programmes of various scales. The mandatory public health and social measures to restrain the extent of the COVID-19 pandemic partially caused the delayed circulation of the non-SARS-CoV-2 respiratory viruses. Therefore, the early post-pandemic seasons may provide an insight into gradual dynamics in the following aftermath of the globally restored respiratory virus circulation scenery. The incidence of the SARS-CoV-2 still requires worldwide attention alongside the influenza viruses, and, lately, respiratory syncytial virus (RSV) has reached the level of global interest to be recommended for systematic surveillance [1,2].

The recent studies on the seasonality patterns (Hong-Kong [3]) and/or the characteristics of the respiratory virus codetections (Germany [4], USA [5]) before and after the COVID-19 pandemic agree to some extent that any distribution and incidence novelties [2,4,6] observed during the pandemic have been temporal [7] and “heterogeneous across locations, populations, and pathogens” [8,9]. Thus, atypical RSV resurgences, both seasonal- and age group-related [9], were already followed by pre-pandemic-like RSV seasonal patterns in 2022–2023 in the United States of America [10] while not normalised yet in the Europe region [11]. The intrigue of the renewed respiratory virus scenery still remains.

On the local scale, in Latvia, the epidemiological data on influenza viruses for the last decade illustrate some perturbations in the influenza seasonality in Latvia. The incidence of the influenza viruses usually reaches epidemic proportions in mid-to-late January and lasts until the spring [12,13]. A different seasonal pattern of the influenza incidence was observed right before the COVID-19 pandemic when the influenza epidemic started in Week 46 with an intensity peak around Christmas 2019 [14]. An insignificant and, presumably, pandemic-related influenza incidence marked the season of 2020–2021, and the delayed start of the influenza epidemic followed in mid-March (Week 10) of the season 2021–2022. The onset of the 2022–2023 epidemic timely resembled the observations of December 2019, but with an incidence unseen since the season 2014–2015 and exceeding [13]. The next influenza season of 2023–2024 reached the epidemic in January (Week 02), which lasted until Week 17, thus following a more characteristic pattern [15].

The simultaneous detection of multiple respiratory viruses by the multiplex real-time quantitative RT-PCR methods (RT-qPCR) enriches the observations on the causes and burden caused by respiratory viruses of worldwide concern and the emergence and/or impact of any locally important targets at any given timeframe. The robustness of the target panels’ composition, the inclusion and summarising criteria of the obtained data, and the longevity of the terminology and the focus used in the analysis are of primary importance for studies regarding respiratory virus coincidence [1,16,17]. Also, the understanding that the codetection of multiple viruses per specimen does not necessarily represent coinfection per patient [16,18,19] may explain gaps between reflections of an individual host scale at a population level [20] as well as observations in cell cultures [21] or animal models [17]. The overall consensus is that a notable but “rather smaller than would be expected” [22] portion of the respiratory virus-positive specimens are codetections or coinfections [20,23,24], mostly occurring in small children [4,22,25,26,27], younger adults (age 14–24 years [24]), or elderly people [28]. More exact conclusions vary regarding the composition and stratification of the datasets, and the data availability on the tested patients’ age may have a more crucial role in addressing possibly hidden bias in any study [20]. Studies on the impact of the codetections and/or coinfections become even more complex for interpretation in the context of the severity of the caused illness and the known or unknown underlying medical conditions. Observations heavily depend on the viruses of the main interest per study and the role of the patients’ age in the clinical manifestation of viral infections [8,29]. As for SARS-CoV-2 infection, the less severe disease among children may be linked to the immune response, the variant of SARS-CoV-2, and its interactions, including exclusions, with other respiratory viruses [30].

Despite the overall limitations of the compatibility of the available information, the data obtained by the multiplex real-time RT-qPCR methods generously illustrate the viral co-circulation and the potential burden on the health system [31]. Globally accumulated data allow to compare the seasonal incidences among regions of a similar climate (Albania, Jordan, Nicaragua, and the Philippines, 2015–2017 [31]) as well as give an understanding of the factors shaping virus interaction, including some knowledge on the affinity and the exclusions of the simultaneously circulating respiratory viruses [20,22,23,24,32].

This study reports observations on the respiratory virus codetections in the respiratory specimens collected from the hospitalised patients and outpatients with symptoms of a respiratory illness during the first season since the pandemic-related measures (social distancing, mandatory use of medical masks, etc.) were lifted in the majority of the public spaces, including nurseries and schools. The data obtained in this study were grouped according to the incidence and the consequent burden of influenza viruses in Latvia. The influenza epidemic occurred 8 December 2022–20 April 2023; the “pre-epidemic” spans the autumn and the beginning of the influenza season (1 September–7 December 2022); and the “post-epidemic” attributes to the late spring and the following summer (21 April–31 August 2023). This study enhances the differences in respiratory virus incidence in terms of the multiple detections among the age groups during three epidemic phases and provides an example of the effect the target composition of the multiplex real-time RT-qPCR panels may have on the conclusions among the different age groups of the patients tested. Our observations have been partially reported in the 34th European Congress of Clinical Microbiology and Infectious Diseases (ESCMID Global 2024) [33].

## 2. Materials and Methods

This retrospective study analysed the data from routine diagnostic tests on acute respiratory infections at the National Microbiology Reference Laboratory of Latvia (NMRL). The laboratory provides diagnostic services for healthcare institutions and implements the epidemiological monitoring programme for respiratory viruses. This study focused on the multi-positive detections per individual specimens analysed by compatible multiplex real-time RT-qPCR panels for detecting of multiple respiratory virus groups.

The dataset represents 4814 respiratory specimens (4754 nasopharyngeal swabs, 48 bronchoalveolar lavages, 12 sputa) submitted from the central (n = 4092) and regional (n = 642) hospitals and sentinel practitioners (n = 80) collected from patients with symptoms of a respiratory illness from 1 September 2022–31 August 2023. Any specimens of non-respiratory material source, as well as duplicate and invalid tests were excluded from the study. Most specimens (n = 3186) were collected during the influenza epidemic period from 8 December 2022 to 20 April 2023, complemented by 787 and 841 specimens of the pre- and post- epidemic periods, respectively (Table 1). 

The specimens were obtained from 2472 female and 2342 male patients, ages 0–100. The female:male (F:M) ratio of the age groups 0–4, 5–14, 15–64, and 65< years was 0.77, 0.87, 0.98, and 1.34, leaning from the male dominance in small children towards the female majority in elders (Table 1). The “age” and “sex” were the only patient data anonymously involved in the study. We do not have any insight on the patients’ background diagnosis, lifestyle factors, or social demographics. The study focuses on the multi-positive detections per specimen and assesses the tendencies of the respiratory virus incidences among the specimens grouped per target, patients’ age, patients’ sex, and/or specimens’ collection date. This study was approved by the Medical and Biomedical Research Ethics Committee of the Riga East Clinical University Hospital, Latvia (Nr.28-A/23, 25 October 2023). The patients’ age for small children up to four years (age 0–4) was also obtained in “months”, thus providing a more exact analysis within the age group. Descriptive statistics as well as the differences among the groups were assessed by the Fisher’s exact test and a nonparametric Mann–Whitney U test, performed with GraphPad Prism 10 for Windows Version 10.1.1—GraphPad Software, LLC.

The laboratory combined various approaches to test the specimens for the possible presence of multiple viruses. From 1 September 2022 to 31 January 2023, 1752 specimens were subjected to the multiplex real-time RT-qPCR by the Anyplex II RV16 Detection panel (V1.07, 01/2018; Seegene, Seoul, Republic of Korea) and tested for the presence of SARS-CoV-2 RNA by the Allplex SARS-CoV-2 Assay (Seegene), the Cobas 6800 (Roche, Basel, Switzerland), the Alinity mSARS-CoV-2 Assay (Abbott, Abbott Park, IL, USA), or the Xpert Xpress SARS-CoV-2/Flu/RSV (Cepheid, Sunnyvale, CA, USA). After 31 January 2023, this approach was replaced by the Allplex Respiratory Panel 3 (V1.07, 09/2019; Seegene) coupled with the Allplex RV Master Assay (V1.07, 09/2019; Seegene) (n = 2326), except for the 66 specimens tested by the Allplex RV Master Assay only. The nucleic acid extraction for these specimens (n = 4144) was performed by NucliSENS easyMAG systemV.2.0 (Biomérieux, Marcy-l’Étoile, France). During the entire period of 1 September 2022–31 August 2023, 670 specimens were tested by an automated system for the nucleic acid extraction and detection by QIAstat-Dx Respiratory SARS-CoV-2 Panel (ref.691214, 03/2020; Qiagen, Shanghai, China) on the QIAstat-Dx Analyzer 1.0 (Qiagen).

All panels provided compatible results for adenovirus (AdV), human bocavirus (BoV), endemic human coronaviruses (CoV-229E, CoV-NL63, and CoV-OC43), influenza viruses (IAV, IVB), human metapneumovirus (MPV), human rhinovirus (RV), and severe acute respiratory syndrome coronavirus 2 (SARS-CoV-2). The differences in the panel composition were addressed, and the more detailed view of the IAV subtypes was generalised, as well as the results obtained of the respiratory syncytial virus subtypes A and B (RSV A and RSV B) and human parainfluenza virus types 1 to 4 (PiV1, PiV2, PiV3, and PiV4), which were summarised as RSV and PiV. In the cases when more exact panels detected the presence of the RNA from both RSV types (n = 3) per specimen, the results were counted as one detection per virus group. No specimens were positive for more than one PiV subtype. In cases where a more detailed view of RSV, PiV, or IAV was needed, we viewed the results accordingly.

The undifferentiated picornavirus group (EV/RV) by the QIAstat-Dx Respiratory SARS-CoV-2 panel was considered an independent detection and was summarised with neither separately detected RV nor enterovirus group (EV). The data on EV circulation were available for five months only (until 31 January 2023) and were not excluded from the dataset for a more informative representation of the results.

The results on human coronavirus HKU1 (CoV-HKU1) (n = 1, female, age group 65<, collected during the first days of the epidemic) had to be disregarded due to the unrepresentative specimen count tested (670/4814). The positive results on bacterial presence (n = 3) and co-presence (n = 1; *Bordetella pertussis*, and BoV, male, age group 15–64, collected three weeks before the end of the epidemic) provided by the QIAstat-Dx Respiratory SARS-CoV-2 panel were excluded from the study.

## 3. Results

The dataset of 4814 respiratory specimens represents patients of all ages, and the data asymmetrically covers four age groups (0–4, 5–14, 15–64, and 65<) (Table 1). Throughout the year (1 September 2022–31 August 2023), 38% of the specimens were detected as positive (1849/4814). The proportion of the positive specimens significantly varied between the individual age groups of the patients (*p* < 0.0001, Fisher’s exact test), with the observed incidence maximum (82%, 521/637) for the age group of the youngest children (age 0–4) declining by each older age group tested (Table 1 and Table A1). The relative risk for single and multiple respiratory virus detection for small children aged 0–4 years stayed significantly higher than for the patients from any other age group independently to the epidemic phases (*p* < 0.05 to *p* < 0.0001, Fisher’s exact test, Table A1). There were no significant differences in the relative risk for the age groups 0–4 and 5–14 between epidemic phases (*p* > 0.05, Fisher’s exact test, Table A2), and the post-epidemic period provided a seasonal decline in respiratory virus incidences for the adult patients only (*p* > 0.0001, Fisher’s exact test, Table A2). We observed no differences in the risk for positivity between the sexes of the patients within any age group during any given timeframe of the epidemic (*p* > 0.05, Fisher’s exact test) (Table A3).

Processing the results according to the compatibility of the multiplex real-time RT-qPCR target panels yielded different counts of the specimens tested per individual virus group with 100% representation of AdV, IAV, IVB, MPV, SARS-CoV-2, and the summarised sets of PiV and RSV, followed (99%, 4748/4814) by BoV, CoV-229E, CoV-NL63, and CoV-OC43, as well as RV (86%, 4144/4814) (Table 2). 

The summarised PiV group represents a half (2392/4814) of the specimens tested for the undifferentiated PiV and a half (2422/4814) tested for four PiV types separately (PiV1, PiV2, PiV3, and PiV4) (Table 2). Only 36% (1752/4814) of the specimens were tested for enteroviruses (EV), with the nonsummable addition of the other 14% (670/4814) of the specimens tested for the undifferentiated picornavirus group (EV/RV) (Table 2).

**Figure 1:**
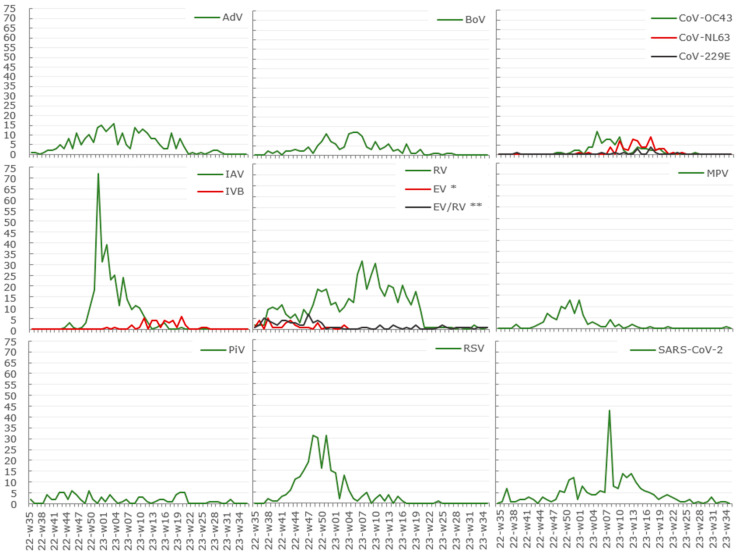
The number of the specimens positive for adenovirus (AdV), bocavirus (BoV), coronviruses (CoV-229E, CoV-NL63, and CoV-OC43), enterovirus (EV), undifferentiated picornavirus group (EV/RV), influenza A virus (IAV), influenza B virus (IVB), metapneumovirus (MPV), parainfluenza virus 1/2/3/4 (PiV), respiratory syncytial virus A/B (RSV), rhinovirus (RV), and severe acute respiratory syndrome coronavirus 2 (SARS-CoV-2) per each week from 1 September 2022 to 31 August 2023. Abbreviations: * EV tested until 31 January 2023; ** EV/RV not tested for the age group 0–4.

The three quarters (75%) of the specimens were received for testing by multiplex real-time RT-qPCR methods during the influenza epidemic and the following month (Week 46 to Week 20), with the mean number of the tested specimens being 156.2 ± SD38.27 per these weeks. Almost two-thirds (61%) were negative for any tested target of the respiratory viruses. This resulted in a significantly higher relative risk (all age groups) for respiratory virus incidence during the pre-epidemic in comparison to the epidemic period (*p* < 0.0001, Fisher’s exact test, Table A2). The maximum of the positives exceeding 50% of the tested specimens per week was observed during the RSV peak, closely followed and overlapped by the IAV peak (Week 46 to Week 52). The notable incidence of positives (49%) returned during the SARS-CoV-2 peak (Week 08). Five virus groups each exceeded the incidence of five percent of the tested specimens (Table 2). These five prevalent virus groups were: RV (12%), IAV (7%), AdV (6%), RSV (5%), and SARS-CoV-2 (5%) (Table 2, Figure 1 and Figure S1). The gap between the next more pronounced virus groups is followed by BoV (3%) and MPV (2%) (Table 2). 

The incidence of RV and SARS-CoV-2 was observed throughout the whole year and peaked during the influenza epidemic, but not simultaneously with the IAV peak (Figure 1). The incidence of AdV peaked alongside IAV (Week 51 to Week 03), and AdV were more scarcely detected throughout the summer (Figure 1). The incidence of BoV was absent in the early autumn, i.e., the beginning of the study period (Week 35 to Week 37), and was limited during the next summer (Figure 1). The endemic coronaviruses (CoV-229E, CoV-NL63, and CoV-OC43) demonstrated a more limited distribution in terms of seasonality, mostly referable to the winter and the following spring (Figure 1). Whereas the pre-epidemic incidence of RSV reached its maximum during the winter’s onset (Week 46 to Week 01) and almost abruptly ended with the decline of influenza epidemic (Figure 1). MPV also were more frequently detected in the autumn and winter, with a small incidence peak from Week 49 to Week 52 (Figure 1). The change of the testing approaches (see Section 2) limited the overview on the incidence of the individual PiV subtypes; therefore, the data on any possible seasonality differences are now blended into the summarised PiV group with a less pronounced incidence during the summer (Figure 1). A notable incidence of EV during the pre-epidemic may not be affected by the onset and peak of IAV (Figure 1). Still, the change of the testing approaches (see Section 2) limits the year’s overview.

**Figure 2:**
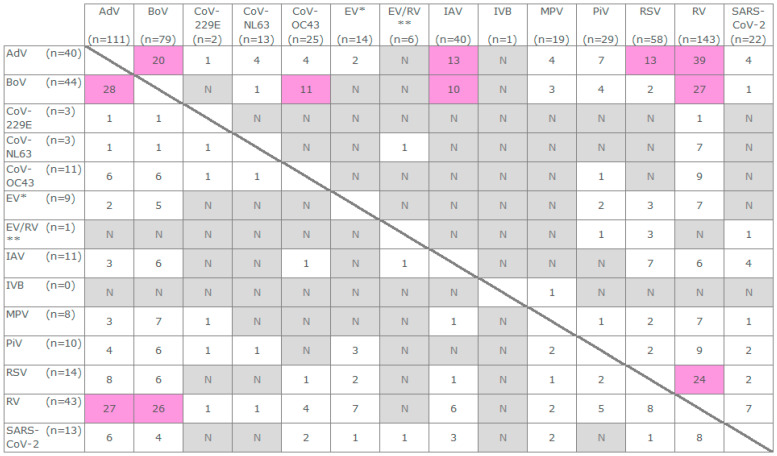
The number of the specimens with detected pairs of viruses in 88 virus combinations of two (upper part) or three and more (bottom part) during 1 September 2022–31 August 2023 (n > 10 painted in pink; negatives marked as gray “N”—not detected). Abbreviations: n = count of the multi-positive specimens (the total sum exceeds the number of the positive specimens since each incidence is recounted per virus involved); * EV tested until 31 January 2023; ** EV/RV undifferentiated picornavirus group (age group 0–4 not tested). A more detailed overview of these combinations is summarised in Table A5.

Almost one-fifth of the positive specimens were identified as codetections (19%, 348/1849) for two or more virus groups, asymmetrically distributed (n = 225, 56, 43, and 24) among the age groups 0–4, 5–14, 15–64, 65<, respectively (Table 1 and Table 2 and Table A4). The mono-positive specimens (1501/1849) were represented by the five dominant virus groups: RV (22%), IAV (18%), SARS-CoV-2 (14%), RSV (12%), and AdV (8%) (Table 2). The virus groups dominating the multi-detections were RV (53%), AdV (43%), BoV (35%), in mutual pairs as well as all three together, followed by RSV (21%), and IAV (15%) (Figure 2 and Table A5). 

The majority of the codetections (65.52%, 228/348) was observed during the influenza epidemic and for the most part represented (77%, 177/228) by children aged 0–4 (n = 136) and 5–14 years (n = 41) (Table 1). The non-epidemic periods were scarcely interpretable in terms of the codetections for adult patients (age 15<), representing in total eleven and six multi-positive specimens during the pre- and post-epidemic (Table 1, Table A2 and Table A4). In total, 88 combinations of 14 virus groups were detected during the entire year (Figure 2 and Table A5). This includes 63 combinations during the influenza epidemic (13 combinations for 48 specimens involved IAV). The pre- and post-epidemic periods were less diverse, with 41 and 18 combinations of 11 and 8 virus groups, respectively (Table A5). The majority (76%, 263/348) of the specimens with codetections were identified as positive for two virus groups (Table 2 and Figure 2). The most pronounced pairs were AdV-RV, BoV-RV, RSV-RV, and AdV-BoV (n = 39, 27, 24, and 20), and the most detected triplets were AdV-BoV-RV (n = 11, patients’ aged 0–14) (Figure 2 and Table A5). Four to five targets were detected in seven multi-positive specimens, all collected from the youngest patients (age group 0–4). These seven specimens were positive for AdV and RV (Table 2 and Table A5).

### 3.1. The Patients’ Median Age

We complemented the comparison of the relative risk regarding incidences per epidemic period (Table A2) with an assessment of the differences between the median age of the patients with the positive and multi-positive specimens collected during the entire year and the epidemic periods. The overall median age of the patients with the specimens positive for at least one virus group was 31 years. It changed significantly (*p* < 0.001, Mann–Whitney) from 12 years during the pre-epidemic to 34.5 and 35 years during the epidemic and the following summer (Table 3). For a more exact analysis for the age group 0–4, we repeated the assessment with the median age in months (0–59) (Table 3), and there were no differences in the median age between the periods of the epidemic (*p* > 0.05, Mann–Whitney).

The patients with multi-positive specimens were younger (3 years) than those with one positive target per specimen (40 years) throughout the entire year (*p* < 0.0001, Mann–Whitney test) as well as each epidemic phase: age 2, 3, and 3 and 28, 44, and 39 years, respectively, in timely order (Table 3). Within the age group 0–4, the children with multi-positive specimens were slightly older (23 versus 20 months; *p* = 0.015, Mann–Whitney), but this difference was not substantiated during the individual epidemic phases (*p* > 0.05) (Table 3). A significant difference in the median age (*p* < 0.05, Mann–Whitney) was observed between the patients with multi-positive specimens during the pre-epidemic and epidemic. Still, there were no differences in the median age between the epidemic phases within the age group 0–4 (*p* > 0.05, Mann–Whitney).

We expanded the assessment of the median age differences of the patients with the positive specimens collected between three epidemic periods for each of the six most dominant virus groups detected—RV, IAV, AdV, RSV, SARS-CoV-2, and BoV (Table 2). The only virus groups with any differences in this regard were RV and SARS-CoV-2. Thus, the median age for patients with RV-positive specimens collected during the pre-epidemic was significantly smaller in comparison to epidemic and post-epidemic phases (3, 16, and 9 years, Table 3) (*p* < 0.0001 and *p* < 0.01, Mann–Whitney). The patients with SARS-CoV-2-positive specimens were also significantly (*p* = 0.0041, Mann–Whitney) younger during the pre-epidemic in comparison to the epidemic—44.5 and 67.5 years (Table 3), but not post-epidemic (*p* > 0.05). We repeated this comparison for multi-positive specimens, and only RV-multi-positives provided one noteworthy difference (pre-epidemic versus epidemic, *p* = 0.023, Mann–Whitney). We also enlarged the RV-positive data pool with the detections for the undifferentiated EV/RV (see Section 2). The median age grew to 13, 23, and 20 years with respective interquartile ranges (IQR) of 2.00–42.25, 3.00–61.00, and 3.00–54.00 for each subsequent epidemic period, and the differences between patients with RV-EV/RV-positive specimens remained true between pre-epidemic and epidemic phases (*p* = 0.0032, Mann–Whitney). Still, the same difference for RV-multi-positives was lost in the RV-EV/RV sum, and the obtained analytical results may reflect the methodological bias.

**Figure 3:**
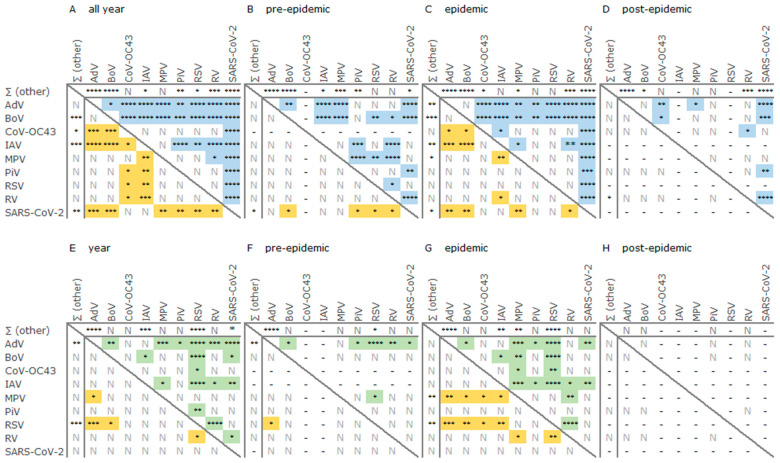
Difference matrices for the median age of the patients whose specimens tested positive per respiratory virus groups during 1 September 2022–31 August 2023 (**A**,**E**), the pre-epidemic (**B**,**F**), the influenza epidemic (**C**,**G**), and the post-epidemic (**D**,**H**). The upper parts of the matrices represent general positivity per target and the bottom parts represent multi-detections. The median age was measured in years (all age groups, **A**–**D**) and months (age group 0–4 years, **E**–**H**). The median age evaluated by the nonparametric Mann–Whitney U test; abbreviations of *p* values: * *p* < 0.05; ** *p* < 0.01; *** *p* < 0.001; **** *p* < 0.0001; N *p* > 0.05; and “-” insufficient case count. Significant differences are coloured in blue and green for positives, and yellow for multi-detections. Abbreviation “∑ (other)” stands for a summarised group of all respiratory virus detections in a given timeframe without the positives for the respective virus group.

We also compared the median age of the patients with positive and multi-positive specimens per nine individual virus groups: AdV, BoV, CoV-OC43, IAV, MPV, PiV, RSV, RV, and SARS-CoV-2. Virus groups with a prevalence of less than four percent of positive specimens per year (Table 2) were excluded from this analysis. The most distinctive median age was observed for patients with AdV, BoV, or SARS-CoV-2-positive specimens (3, 2, and 65 years, Table 3). Their median age was significantly different (*p* < 0.05 to *p* < 0.0001, Mann–Whitney) in comparison to each of the other eight individual virus groups during the year (Figure 3A). The groups of IAV and RV-positives (34.5 and 9 years) had six and five significant differences (Table 3 and Figure 3A). The patients’ median age was undistinguishable between groups of the MPV, PiV, and RSV-positive detections, and each of these groups yielded four significant differences with the other four already mentioned more age-specific virus groups. A less specific median age was observed for the patients with CoV-OC43-positive specimens (Figure 3A). Overall, the difference matrices of the year and each of the epidemic phases were not identical (Figure 3A–D, upper matrices); nevertheless, the significant differences between the patients’ median age of AdV, BoV, or SARS-CoV-2-positives remained.

The median age of the patients with multi- and mono-positive specimens throughout the year and influenza epidemic was significantly different for the nine individual virus groups taken as the centre of focus (*p* < 0.05 to *p* < 0.0001 Mann–Whitney, Table 3). The observation complements the significantly high relative risk of the codetections among children (*p* < 0.0001, Fisher’s exact test, Table A1). We continued by comparing the patients’ median age with multi-positive specimens of the nine more prevalent virus groups. The patients with the IAV, SARS-CoV-2, or CoV-OC43-positive codetections had a higher median age (7, 9, and 7 years) than the groups of the patients with RV, AdV, BoV, RSV, MPV, or PiV-multi-detections (2 to 3 years, Table 3) (*p* < 0.05 to *p* < 0.0001, Mann–Whitney, Figure 3A–D, bottom matrices), with the exception of no differences (*p* > 0.05) between the groups of the MPV and CoV-OC43-multi-positives (Table 3 and Figure 3A–D, bottom matrices).

We repeated the same assessment of the differences in the median age of the youngest patients, age group 0–4 (0–59 months). The most distinctive median age throughout the year was observed for the AdV and RSV-positives (29 and 11 months, Table 3). Each provided six significant differences (*p* < 0.05 to *p* < 0.0001, Mann–Whitney, Figure 3E) with the other eight virus groups (Figure 3E). The next in rank was the group of the IAV-positives (30 months, Table 3) with five significant differences (Figure 3E). The group of the AdV-positives dominated the difference matrix of the pre-epidemic, followed by the RSV-positives (five and two differences, Figure 3F). The small children with the RSV, MPV, or SARS-CoV-2-positive specimens had the most minor median age (6, 7, and 12 months) during the epidemic (Table 3), significantly different in comparison to groups of AdV or IAV-positives with the highest median age (27.5 and 30 months; *p* < 0.05 to *p* < 0.0001, Mann–Whitney, Figure 3G). The most distinctive median age was observed for the IAV-positives (six differences), followed by the groups of AdV, RSV, and MPV (five differences, Figure 3G). 

### 3.2. Characteristics of the Multi-Positive Results per Virus Groups

In this chapter, we give a more focused and detailed glance at the codetections and some highlights of the results for each virus group, arranged in order of the prevalence in the data pool of multi-positive detections. 

#### 3.2.1. Rhinovirus

RV was detectable (n = 511) during the entire year in 36 combinations with 11 other tested virus groups (n = 186) (Figure 1 and Figure 2 and Figure S1, Table 2 and Table A5) as well as alone (n = 325), dominating the positive results in comparison to other respiratory virus groups observed in the study even though RV was tested for only 86% of all specimens (Table 2). We did not interpret the positive results for the undifferentiated picornaviruses (EV/RV) (Table 2). The majority (80%) of the RV-positive multi-detections involved AdV, BoV, and RSV, followed by SARS-CoV-2, EV, PiV, CoV-OC43, IAV, MPV, CoV-NL63, and/or CoV-229E (Figure 2 and Table A5). The overall median age for all patients with the RV-positive codetections was 2 years (Table 3). Throughout the entire year, the specimens with the RV-mono detections represented significantly older patients (median 32 years; *p* < 0.0001, Mann–Whitney), and this difference remained significant between all three epidemic periods (*p* < 0.001, Mann–Whitney) (Table 3). Whereas, for the age group of children aged 0–4 years (0–59 months), the representatives of RV-multi-positive specimens had a higher median age in comparison to the RV-mono-positives throughout the entire year and pre-epidemic (23 and 26 versus 19 and 10 months; *p* < 0.05, Mann–Whitney) (Table 3). 

#### 3.2.2. Adenovirus

We observed 274 AdV-positive detections throughout the year with a limited incidence during post-epidemic (Figure 1 and Figure S1). AdV, the second most frequent former of the codetections (n = 151), was less often (n = 123) detected solo (Table 2 and Table A4), but in 33 combinations with RV, BoV, RSV, IAV, CoV-NL63, PiV, CoV-OC43, SARS-CoV-2, MPV, CoV-NL63, EV, and/or CoV-229E (Figure 2 and Table A5). The differences in the overall median age for all patients with the AdV-positive codetections (2 years) and mono-detections (6 years) were significant during the entire year (*p* < 0.0001, Mann–Whitney) as well as the influenza epidemic (2 and 6 years, *p* < 0.0001) and the post-epidemic (2.5 and 13.5 years, *p* < 0.05) (Table 3). However, the median age of the small children (29 months) was indistinguishable in terms of this context (*p* > 0.05) (Table 3). The majority (82%, 225/274) of the AdV-positive specimens were referable to the age groups 0–4 and 5–14 (Table A5). Also, three quarters (74%, 111/151) of the specimens with AdV-positive-codetections were collected from the patients aged 0–4 years, representing 67% (55/111) of the AdV-positives per this age group (Table 2, Table A4 and Table A5). The median age of the patients (all age groups) with AdV-positive specimens (3 years, Table 3) was significantly smaller (*p* < 0.0001, Mann–Whitney) than the median age of patients with AdV-negative respiratory virus detections (39 years, IQR 6.00–67.00) during the year as well as all three epidemic phases (*p* < 0.0001; median age 23, 43, and 40 years (IQR—2.00–58.00, 7.00–69.00, and 18.50–65.00) for AdV-negatives in timely order) (Figure 3A–D). The median age of the small children (age group 0–4, 0–59 months) with AdV-positive and multi-positive specimens throughout the year was 29 months and a little higher (32.5 and 30 months) during pre- and epidemic (Table 3). The median age of the children with AdV-negative respiratory virus detections was significantly lower throughout the year, pre-epidemic and epidemic (19, 19, and 18.5 months; IQR—5.00–35.00, 4.50–33.00, and 5.00–35.00; *p* < 0.0001, Mann–Whitney). This difference was also significant for the codetections throughout the year and pre-epidemic: 29 and 30 months (Table 3) versus 20.5 and 20 months (IQR—7.00–33.25, 5.75–33.50; *p* < 0.05, Mann–Whitney).

#### 3.2.3. Bocavirus

Almost all specimens (99%), with an exception of 66 (n = 65 collected Week 02), were tested for BoV (Table 2). A slight incidence peak with up to twelve BoV-positive specimens per week (mean 8.3 ± SD3.34) providing almost a half of all BoV-positive detections (76/157) was observed during Week 51 to Week 07 (Figure 1 and Figure S1). 3% of the tested specimens were BoV-positive (157/4748) (Table 2). BoV was the sixth most often detected virus group and the third most frequent former of the codetections (Table 2). Three quarters (78%) of the BoV-positives contributed to 34 combinations with other eleven respiratory virus groups, heavily dominated by RV and/or AdV (82% of the BoV-multi-positives), followed by CoV-OC43, IAV, MPV, PiV, RSV, EV, SARS-CoV-2, CoV-NL63, and/or CoV-229E (Figure 2 and Table A5). A high prevalence (68%, 107/157) of the BoV-positives was observed for the age group 0–4 (Table 2 and Table A4), and the relative risk for the BoV-positive detection for this age group was 2.98 (95%CI 1.90 ÷ 4.71) in comparison to the next closest one age group 5–14 (*p* < 0.001, Fisher’s exact test). Consequently, the median age of the patients (all age groups) with BoV-positive specimens was significantly lower than observed for any other of the eight most prevalent virus groups throughout the year (*p* < 0.05 to *p* < 0.0001, Mann–Whitney, Figure 3A) and partially during the epidemic phases (Figure 3C,D). This difference remained significant between the BoV-positives (all age groups, Table 3) against a summarised group of the BoV-negative respiratory virus detections (n = 1671) throughout the year and each epidemic phase (35, 17, 40, and 36 years; IQR—5.00–66.00, 2.00–54.00, 6.00–68.00, and 7.00–62.00; *p* < 0.0001, Mann–Whitney) (Figure 3A–D). Whereas, among the youngest patients (aged 0–4 years, 0–59 months), the median age of BoV-positives or multi-positives was distinctive only in comparison to some of the other eight more prevalent virus groups and not the summarised groups of the BoV-negatives (*p* > 0.05, Mann–Whitney) (Figure 3E–H). Thus, the median age of the small children with BoV-positive specimens (22 months, Table 3) was significantly different (*p* < 0.05 to *p* < 0.0001, Mann–Whitney) from the smaller or higher median age of the respective groups of RSV and SARS-CoV-2 (11 and 12 months) or AdV and IAV (29 and 30 months) throughout the year (Table 3 and Figure 3E). As well as the same tendency was pronounced in comparison to the RSV and MPV (6 and 7 months) or AdV and IAV (27.5 and 30 months) throughout the epidemic (Table 3 and Figure 3G). This difference remained significant for multi-positives only in comparison to the RSV (year and epidemic, Figure 3E,G) and MPV (epidemic, Figure 3G). We also observed the differences in the median age for patients with the BoV-multi- and mono-positive specimens. Thus, the median age of the patients (all age groups) with BoV-multi-positives was significantly smaller throughout the year and influenza epidemic (2 versus 7 and 13 years, *p* < 0.01, Mann–Whitney) (Table 3). In contrast, the median age of the children (age group 0–4) with BoV-multi-positive specimens was significantly higher (22 versus 11 months, *p* < 0.05, Mann–Whitney) and only during the epidemic (Table 3). Among the virus groups exceeding 2% of the positivity per at least 86% of all tested specimens, BoV stood out in terms of the F:M ratio. The BoV-positive patients had the most minor F:M ratio (0.65; Table 2) with a relative risk of 1.62 (95%CI 1.18 ÷ 2.21) for male patients (all age groups) to have the BoV-positive detection (*p* = 0.004, Fisher’s exact test). The F:M ratio appeared biased towards the male proportion in each age group (Table A4); nevertheless, there were no attributable differences in the relative risk per age group (*p* > 0.05, Fisher’s exact test).

#### 3.2.4. Respiratory Syncytial Virus

The fourth per total count of the codetections was the group of the RSV-summarised (n = 72), representing sixty RSV B, eight RSV A, and six RSV-undifferentiated, altogether spanning more than twenty combinations with RV, AdV, BoV, IAV, EV, PiV (PiV 1, PiV 2, PiV 4), SARS-CoV-2, MPV, EV/RV, and/or CoV-OC43 (Table 2 and Table A5, Figure 2). Three specimens were positive for both (RSV A and RSV B) types: two of them in combination with AdV (age group 0–4, female and male, collected during the late pre-epidemic) and one without any additional targets detected (age group 65<, male, collected at the peak-start of the influenza epidemic). Almost all 259 RSV-positives were detected during the pre-epidemic and epidemic, except for one RSV-mono-detection post-epidemic (Figure 1 and Figure S1). Three-quarters of all RSV-positives (75%, 194/259) and codetections (76%, 55/72) emerged as a wave-like maximum from November 2022 to the beginning of January 2023 (Figure 1), yielding 11 to 31 RSV-positive specimens per week (mean 19.4 ± SD8.07). The relative risk for the RSV-positive detection (3.63, 95%CI 2.88 ÷ 4.57; *p* < 0.0001, Fisher’s exact test) and codetection (1.56, 95%CI 1.05 ÷ 2.33; *p* < 0.05) during the pre-epidemic was significantly higher than the epidemic. RSV was observed as the third most frequent virus group. RSV affected the youngest and the oldest patients (age groups 0–4 and 65<). The notable RSV incidence for the age group 65< provided 31% of all RSV-positives (81/259), but only eight of all RSV-positive codetections (Table A4). Nevertheless, the positivity observed for the patients’ age group 0–4 was significantly higher (relative risk 4.54, 95%CI 3.50 ÷ 5.88, *p* < 0.001, Fisher’s exact test). This unequal incidence of the RSV-multi- and mono-detections among the age groups echoed as the significantly smaller overall median age for the patients with the RSV-multi-positive specimens throughout the year (2 versus 47 years, *p* < 0.001, Mann–Whitney) as well as each of the both relevant epidemic phases (Table 3). The specimens collected from the patients aged 0–4 were indistinguishable in this regard (*p* > 0.05) (Table 3). The incidence peak for RSV-positives spans the weeks of pre-epidemic and epidemic (Figure 1). Consequently, the differences in the median age of patients with RSV-positives between both epidemic phases were insignificant (*p* > 0.05, Mann–Whitney). Also, the median age of RSV-positives (19 years, all age groups) was significantly smaller than observed for the summarised group of the RSV-negative respiratory virus detections (n = 1590) throughout the year (32 years, IQR 4.00–62.00; *p* < 0.0001, Mann–Whitney), but not individual epidemic phases (Figure 3A–D). Within the age group 0–4, these differences were significant either throughout the year or both epidemic phases (*p* < 0.05 to *p* < 0.0001, Mann–Whitney) (Figure 3E–H). Namely, the median age for children with RSV-negative detections was higher (23, 26.5, and 22 months with IQR—12.00–38.75, 10.75–42.00, and 12.00–38.00, respectively). In comparison with the other eight virus groups, the 76% incidence of RSV-positives in the marginal age groups (0–4 and 65<, Table A4) smoothed out the overall median age (all age groups) throughout the year and epidemic (Table 3). Overall, the median age was significantly higher than observed for the groups of AdV and BoV and significantly lower than for SARS-CoV-2-positives (Table 3, Figure 3A–C), with some nuances per period (*p* > 0.05 to *p* < 0.0001, Mann–Whitney). Similarly, for the multi-positives (all age groups), the difference in the median age was significant between the groups of the CoV-OC43 (*p* < 0.05), IAV, or SARS-CoV-2 (*p* < 0.01) positive codetections (Figure 3A). Whereas for the patients of the age group 0–4, such comparison provided six and five significant out of eight possible differences throughout the year and epidemic (Figure 3E,G), and the small median age of RSV-positives was also significantly pronounced in contrast to the median age of the patients with RSV-negative respiratory virus detections (Figure 3E–G). Also, the second most minor median age characterised the specimens with the RSV-positive codetections (17.5 months) (Table 3), especially during the epidemic (9.5 months), providing a significant difference in the median age with the RSV-negative codetection representatives (26 and 23 months, IQR 16.00–38.00 and 16.00–36.00; *p* < 0.001, Mann–Whitney) (Figure 3E,G). The tendency for the RSV-positive codetections within the age group remained pronounced in comparison to the sets of the AdV (*p* < 0.001), BoV, IAV (*p* < 0.01), and/or CoV-OC43, RV-positive codetections (*p* < 0.05), but not for groups of MPV and PiV-multi-positives (*p* > 0.05) during the epidemic (Figure 3G). 

#### 3.2.5. Influenza Viruses

The fifth most pronounced group of the codetections was IAV-positives (n = 51) (Table 2), representing 13 combinations with RV, BoV, SARS-CoV-2, AdV, RSV, CoV-OC43, EV/RV, and/or MPV (Figure 2 and Table A5). One IVB-positive codetection (in combination with MPV, male, age group 0–4, collected Week 01) (Table A4 and Table A5). It was not summarised with IAV-positives. The analytical approaches provided 100% coverage of the specimens for the differentiated identification of IAV and IVB (Table 2). The incidence of IVB (n = 41) followed the peak of IAV and lasted past the end of the influenza epidemic (Figure 1 and Figure S1). The incidence of the IAV (n = 322) dominated both groups of the influenza viruses (relative risk 7.85, 95%CI 5.70 ÷ 10.83, *p* < 0.0001, Fisher’s exact test). A more precise analysis by one of the target panels (14% of the specimens tested, patients age 15<) provided an observation of the similar incidence of two IAV subtypes: H1N1/2009 and H3 (16 and 20 positives during Week 43 to Week 13). Three of these positives were codetections: one H1N1/2009 in combination with AdV (female, age group 15–64, collected during the first days of the epidemic) and two H3-positives (one in combination with RSV, male, age group 15–64; the other with SARS-CoV-2, female, age group 65<; both collected early December 2022). The maximum number of IAV-positives (128/322) was detected in the age group 15–64; nevertheless, the presence of the IAV-positive specimens was highly pronounced (21%) in the age group 5–14 (median age 7 years), significantly exceeding (*p* < 0.01, Fisher’s exact test) the incidence per any other age group (Table A4). The overall median age of the patients with the IAV-positives and codetections was significantly higher (34.5 and 7 years) than observed for the summarised group of IAV-negative respiratory virus detections and multi-positives (31 and 2 years, IQR—3.00–64.00, 1.00–7.00; *p* < 0.05 and *p* < 0.001, Mann–Whitney) as well as the groups of the AdV, BoV, PiV, RSV, and RV-positives and multi-positives throughout the year (*p* < 0.01 to *p* < 0.0001, Mann–Whitney) (Table 3 and Figure 3A). The difference matrices for pre-epidemic and epidemic (all age groups) with some variations still highlighted the IAV groups as the most different from the respective groups of AdV and BoV in terms of median age (Figure 3A–D). As for the age group 0–4, we observed six out of eight possible differences between the median age of the IAV-positives compared to other virus groups (Figure 3G) during the epidemic. The median age of children with IAV-positive specimens was significantly higher than observed for the BoV, MPV, PiV, RSV, RV, and SARS-CoV-2-positives (30 versus 6 to 22 months, Table 3) and the summarised group of IAV-negative respiratory virus detections during the epidemic (20 months, IQR 7.00–34.25) (*p* < 0.05 to *p* < 0.0001, Mann–Whitney) (Figure 3G). Whereas for IAV-multi-positives, the tendency remained pronounced in comparison to the RSV and MPV-positive codetections (27 versus 7 and 10 months) (*p* < 0.01 and *p* < 0.05, Mann- Whitney) and there were no differences in the patients’ median age for children representing IAV-positive and IAV-negative codetections (*p* > 0.05, Mann–Whitney) (Table 3 and Figure 3G). 

#### 3.2.6. Parainfluenza Virus

The modest PiV-positivity per all tested specimens (1.8%, 88/4814) was overcome by a high proportion of the detectability as a part of the virus combinations (44%, 39/88) (Table 2 and Figure 2), noticeably represented by the youngest patients aged 0–4 years (30/39) (Table A4), especially during the pre-epidemic (16/19). Detailed recognition of the PiV types 1 to 4 was provided for half of the specimens, thus the summarised group of 39 PiV-positive codetections in 19 combinations with BoV, RV, AdV, EV, MPV, RSV, EV/RV, CoV-229E, CoV-NL63, CoV-OC43, and/or SARS-CoV-2 was formed by eleven PiV-undifferentiated, eleven PiV 4, nine PiV 1, five PiV 3, and three PiV 2 (Table A5). The summarised data of the PiV weekly incidence limits any conclusions on the PiV subgroup seasonality (Figure 1 and Figure S1). The median age of the patients (all age groups) with PiV-positive specimens was smaller (*p* < 0.01, Mann–Whitney) than observed for the summarised group of PiV-negative respiratory virus detections throughout the year and pre-epidemic (6 and 3, Table 3, versus 32 and 17 years, IQR 4.00–64.00 and 2.00–54.00) (Figure 3A,B). The median age of the PiV-positive groups was also smaller in comparison to the groups of SARS-CoV-2 and IAV-positives (*p* < 0.001 to *p* < 0.0001, Mann–Whitney), but exceeded the respective median age of the AdV and BoV groups (*p* < 0.01 to *p* < 0.001, Mann–Whitney) (Table 3 and Figure 3A–D). The incidence of the PiV-multi-positives in the age group 0–4 was reflected as the significantly smaller median age of the patients (all age groups) with the PiV-multi-positives in contrast to the groups of PiV-mono-detections throughout the year and each epidemic phase (*p* < 0.05 to *p* < 0.0001, Mann–Whitney) (Table 3). Consequently, the median age of the patients with PiV-multi-positive specimens was also significantly smaller than observed for the groups of SARS-CoV-2, CoV-OC43, and IAV-multi-positives (Table 3 and Figure 3A,B). The median age of children (age group 0–4) with PiV-positive specimens was significantly lower than observed for the AdV group throughout the year, pre-epidemic and epidemic (Table 3 and Figure 3D–G), but exceeded the median age of RSV-positives (year). Nevertheless, we observed no significance in terms of the median age for children aged 0–4 with the PiV-multi-positive codetections (Figure 3D–H).

#### 3.2.7. Coronavirus OC43

The positive detections of CoV-OC43 were mostly (87%) observed during the epidemic (Figure 1 and Figure S1), with a maximum from Week 03 to Week 10 (mean 8 ± SD2.45 cases per week) yielding 56% of all CoV-OC43-positives and 61% of all codetections per target. Almost half (42%, 36/85) of all CoV-OC43 detections were in 11 combinations with AdV, RV, BoV, SARS-CoV-2, CoV-229E, CoV-NL63, IAV, PiV, and/or RSV (Table 2, Figure 2, and Table A5). More than a half of the CoV-OC43-positives were representatives of the adult patients (51/85 for age groups 15–64 and 65<). Also, a half of the codetections (47%) were represented by the age group 0–4, but another third 31%—by adults age 15–64 (Table A4). This notably high proportion of the multi-positives for the adult patients (age group 15–64) was a distinctive characteristic of CoV-OC43. It was illustrated by the higher median age (2 years for all age groups) for patients with CoV-OC43-multi-positive specimens than observed for the summarised group CoV-OC43-negative respiratory virus codetections (7 years, IQR 1.00–7.00; *p* < 0.05, Mann–Whitney) and six out of eight virus groups throughout the year (Figure 3A). Nevertheless, during the influenza epidemic, i.e., the more relevant weeks for the CoV-OC43 incidence, this difference was only partially pronounced. Namely, the median age of the patients with CoV-OC43-positives and multi-positives (25 and 4.5 years, all age groups, Table 3) was distinctively higher than observed for AdV and BoV-positives (3 and 2 years; *p* < 0.0001, Mann–Whitney) and multi-positives (2 years both, *p* < 0.05, Mann–Whitney), and also lower than observed for SARS-CoV-2 or IAV-positives and the summarised group of the CoV-OC43-negative detections (67.5, 33.5, and 36 years, IQR 4.00–64.00; *p* < 0.05 to *p* < 0.0001, Mann–Whitney) (Figure 3C). Within the age group 0–4, the median age of CoV-OC43-positives was significantly higher than observed for RSV and MPV-positives and multi-positives (22 versus 6 to 10 months, *p* < 0.05 to *p* < 0.01, Mann–Whitney) (Table 3 and Figure 3C).

#### 3.2.8. SARS-CoV-2

For SARS-CoV-2, the fifth in the rank of the positive specimens, only 14% (35/250) were codetections (Table 2); it was the lowest ratio of the codetections among all other virus groups with the overall positivity exceeding 2% of the tested specimens. SARS-CoV-2 contributed in 19 combinations with RV, AdV, BoV, IAV, MPV, CoV-OC43, RSV, EV, and/or PiV, with the second highest rate of 1.8 specimens per combination within the dataset (Figure 2 and Table A5). A rather uniform incidence of SARS-CoV-2 was observed in our dataset throughout the year, with a higher positivity during the influenza epidemic and, especially, Week 8 (Figure 1 and Figure S1). Half of all SARS-CoV-2-positives (51%) were detected in the specimens collected from the oldest patients (age group 65<) (Table A4). Also, the highest incidence per age group was observed in comparison to the age groups 0–4, 5–14, 15–64 (relative risk 1.60, 95%CI 1.08 ÷ 2.37, *p* < 0.05; 3.23, 95%CI 1.63 ÷ 6.47, *p* < 0.001; and 1.76, 95%CI 1.35 ÷ 2.30, *p* < 0.001, Fisher’s exact test). Moreover, the highest observed incidence for any tested respiratory virus within the age group 65< was mirrored as the highest median age in comparison to other eight virus groups as well as the summarised group of SARS-CoV-2-negatives (23 and 27 years, IQR 3.00–59.00 and 4.00–62.00) throughout the year and epidemic (*p* < 0.01 to *p* < 0.0001, Mann–Whitney). The differences with the groups of AdV, BoV, PiV, RV (Table 3), and SARS-CoV-2-negatives (10 and 30 years, IQR 2.00–51.00 and 5.00–57.50) remained significant during pre- and post-epidemic (Figure 3A–D). The overall median age (9 years, Table 3) for the patients with the SARS-CoV-2-multi-positive detections also stayed higher (*p* < 0.05 to *p* < 0.001 Mann–Whitney) than observed for most of the other viruses tested with the exception of IAV and CoV-OC43 throughout the year (Figure 3A) and more exceptions during the pre-epidemic and epidemic. Within the age group 0–4, the median age of the patients with SARS-CoV-2-positive specimens was significantly lower than observed for AdV, BoV, IAV, and RV-positives throughout the year (*p* < 0.05 to *p* < 0.0001, Mann–Whitney) (Table 3 and Figure 3E). The median age was smaller in comparison to the group of AdV-positives also during pre- and epidemic (16 and 12 versus 32.5 and 27.5 months, *p* < 0.05 and *p* < 0.01, Mann–Whitney) and smaller in comparison to the IAV-positives (30 months, *p* < 0.01, Mann–Whitney) during the epidemic. There were no significant observations regarding the median age in SARS-CoV-2-multi-positives collected from the age group 0–4 (Figure 3).

#### 3.2.9. Metapneumovirus 

The main incidence of the MPV was observed from November to January (71%, Weeks 46–02, mean 8.22 ± SD3.27 detections per week) (Figure 1 and Figure S1), also yielding 70% of all MPV-positive codetections. MPV was co-detected in 15 combinations with BoV, AdV, PiV, RV, SARS-CoV-2, RSV, CoV-229E, IAV, and/or IVB (Figure 2 and Table A5). The MPV-positive specimens (n = 105) were characteristic to all four age groups (median age 34 to 50 years, throughout the year or individual epidemic phases) (Table 3), with a higher incidence (5.5%, 35/637, Table A4) within the age group of small children (age 0–4). Also, the majority of the MPV-multi-positives (70%, 19/27) were attributable to the same age group, and there were no cases of the MPV-multi-positives in the specimens collected from the patients of the age group 65< (Table A4). Consequently, the median age of the patients with multi-positive specimens was significantly smaller (2 years) than observed for MPV-mono-positives throughout the year, pre-epidemic and epidemic (59, 59, and 51 years, *p* < 0.001 to *p* < 0.0001, Mann–Whitney) (Table 3). Also, within the whole dataset (all age groups), the median age of the patients with MPV-multi-positive specimens was smaller in comparison to the groups of SARS-CoV-2 and IAV-multi-positives during the epidemic and the entire year (Figure 3A,C). Zero MPV-multi-positives were detected post-epidemic (Table 3 and Table A4). Within the age group of the small children (age 0–4), the small median age (7 months) of the patients with MPV-positive specimens (Table 3) was significantly different from the summarised group of MPV-negative respiratory virus detections (21 months, IQR 11.00–37.50) and five out of eight individual groups of AdV, BoV, CoV-OC43, IAV, and RV-positives throughout the epidemic (*p* < 0.05 to *p* < 0.001, Mann–Whitney) (Table 3 and Figure 3G). These six differences remained significant (*p* < 0.05 to *p* < 0.01, Mann–Whitney) also for MPV-multi-positives (Table 3 and Figure 3G), including the summarised group of MPV-negative multi-positives (19 months, IQR 5.00–37.00). Another feature of the MPV-positives was the skewed F:M ratio towards higher incidence in female patients (all age groups, relative risk 1.67, 95%CI 1.13 ÷ 2.47, *p* < 0.05, Fisher’s exact test), actually represented by the age group 65< (relative risk 3.28, 95%CI 1.29 ÷ 8.33, *p* < 0.05, Fisher’s exact test), since in the other age groups the skewed F:M ratio (Table 2 and Table A4) did not withstand the relative risk calculation (*p* > 0.05, Fisher’s exact test).

#### 3.2.10. Enterovirus

Data on EV were obtained during five months, i.e., the autumn of 2022 and the first seven weeks of the influenza epidemic (1 September 2022–31 January 2023). Thus, the results represent half (55%) of all specimens collected from children (age 0–14) and one-third (31%) of the adult patients (age 15<) (see Methods; Figure 1 and Figure S1, Table 2 and Table A4). Almost all of the EV-positives (29/32) and multi-positives (21/23) were attributable to the youngest patients (age group 0–4) (Table A4 and Table 3). The majority (72%, 23/32) of the EV-positives were codetections in eleven combinations with RV, BoV, PiV, AdV, and/or RSV (Figure 2, Table A4 and Table A5). 

#### 3.2.11. Coronavirus NL63 

CoV-NL63 was detected (n = 60) during the first semester of 2023 (Figure 1), mostly (93%, 56/60) from Week 08 to Week 20 with up to nine detections per week (mean 4.31 ± SD2.66). Seven combinations of 16 CoV-NL63-positive codetections were formed with AdV, BoV, RV, EV/RV, CoV-229E, CoV-OC43, and/or PiV (Table 2 and Table A5, Figure 2). Half of the codetections were represented by the specimens collected from the patients of age 0–4 (Table A4), providing a distinctive overall median age in contrast to CoV-NL63-mono-positives (4.5 and 40 years, *p* < 0.01, Mann–Whitney) (Table 3). Within the age group age 0–4, the median age of the eight patients with CoV-NL63-multi-positive specimens was significantly higher (50 months) than observed for five CoV-NL63-mono-positives (19 months, *p* < 0.05, Mann–Whitney) (Table 3 and Table A4).

#### 3.2.12. Coronavirus 229E 

Five of 13 positives for CoV-229E were codetections in five combinations with AdV, RV, BoV, CoV-NL63, CoV-OC43, MPV and/or PiV (Table 2 and Table A5, Figure 2). Half of the positives and codetections (7/13 and 3/5) were observed in April (Week 03 to Week 17, Figure 1 and Figure S1). The incidence of the CoV-229E-positive detections was unevenly scattered among the age groups and one positive detection for patients aged 0–4 (Table A4).

## 4. Discussion

The advantage of the multiplex real-time RT-qPCR methods in routine diagnostics and respiratory virus monitoring is the ability to characterise the codetection pattern of the respiratory viruses. Along with the influenza viruses (IAV and IVB), this study analysed the co-incidence of AdV, BoV, MPV, PiV, RSV, EV, RV, CoV-229E, CoV-NL63, CoV-OC43, and SARS-CoV-2 among the patients with the symptoms of an acute respiratory illness mostly (98%) sampled for analysis in central and regional hospitals during 1 September 2022–31 August 2023 in Latvia. The diagnostic testing was performed at the National Microbiology Reference Laboratory of Latvia (NMRL). The specimens were grouped according to the patient’s age and sex and interpreted according to the timeline of the influenza epidemic in Latvia.

The incidence of influenza viruses (363 positives—322 and 41 for IAV and IVB) is the main interest of the monitoring programmes but was the second highest in terms of this dataset, preceded by RV and followed by AdV, RSV, and SARS-CoV-2 (Table 2). Our focus on the specimens positive for more than one virus group changed the leaderboard. The most frequent participants in the combinations of the codetections per all age groups within each epidemic period were RV and AdV, accompanied by RSV (mainly during the pre-epidemic) and BoV (mostly during the epidemic and after that) (Table A4). AdV, BoV, and RV (detected in pairs and triplets) lead the overall landscape of the codetections throughout the entire year (Figure 2 and Table A5). These four virus groups have already been observed as the most prevalent codetections in Central Europe ten years preceding the COVID-19 pandemic [23], with the mutual affinity-exclusion scores characterised as closer to affinity to a very moderate extent [23]. The positive associations between AdV and BoV have also been reported in France (2018–2020, all age groups [34]) with dissonant observations regarding RSV and RV (actually, EV/RV undifferentiated). RSV, RV, and AdV have been characterised as the lead participants in BoV-positive codetections also in Spain (2005–2013, children age 0–14 [35]) and Croatia (timescale unclear, children age 0–18 [36]), while AdV was absent from this top three of the BoV-positive codetections in Norway (2006–2017, age 0–16 years [18]), perhaps due to the different target composition of the testing method. The pairs of AdV-RV, RSV-RV, and BoV-RV were the most common combinations in the hospitalised children also across the continent, in Korea (2015–2019), whereas the codetections of influenza viruses with RV or endemic coronaviruses were more pronounced in the age group 65< [28]. As for the age group 65< in our dataset, more than half of the codetections (13/24) involved RV (five, four, and three in pairs with SARS-CoV-2, RSV, or BoV), and the next in rank was SARS-CoV-2 with ten codetections, half of them in the already mentioned pairs with RV (Table A4 and Table A5). 

BoV, AdV, and EV were the only ones more frequently detected as a part of the multi-positives (78%, 123/157; 55%, 151/274; 72%, 23/32) (Table 2). These proportions were even higher in the specimens collected from the patients aged 0–18 (85%, 63%, and 73%) (Table A4). Our data are concordant with pre-pandemic observations in Spain (2005–2013) and Norway (2006–2017), where 75% and 83% of the BoV-positives were codetections (hospitalised children [18,35]). A study of primary care patients in France shortly before the onset of the pandemic (2018–2020, all age groups) reported all BoV-positives (n = 12) as codetections, mostly (10/12) in the specimens collected from the children aged 0–5 years [34]. Similarly, also among children, the proportion of AdV-positive codetections exceeded AdV-mono-positives in New York, Northeast US (2021–2022 [22]), and Morocco (2021 [37]). The high EV incidence in the multi-positive detections observed in our study during the limited timespan of five months is comparable to the observations from the data of eleven years in Norway [18] and suggests that even the incomplete and uneven target coverage for the tested specimens in the case of EV may be of some advantage.

The quite opposite tendency was observed for IAV, SARS-CoV-2, and RSV, which were mainly detected alone (271/322, 215/250, 187/259) as well as mostly shaped the overall incidence of the respiratory virus groups in our dataset (Table 2 and Table A4, Figure 1 and Figure S1). Moreover, only three and seven SARS-CoV-2-positive RSV and IAV codetections and no triplets of these three viruses were obtained (Table A5). The majority of the RSV incidence happened during the autumnal onset of the SARS-CoV-2 detections and the first weeks of the influenza epidemic, but the peak of the SARS-CoV-2 incidence (Week 08; 43 positives; including one codetection with RV) followed the decrease in IAV-positives (Figure 1 and Figure S1). This incidence pattern may agree with the studies on the actual coinfections of SARS-CoV-2 in cell cultures as well as the analysis of the clinical and surveillance data reporting the presence of IAV [38], RSV [7,32], or IAV and RSV [21] or other respiratory viruses [22] as the limiters for SARS-CoV-2 activity. Also, higher SARS-CoV-2 and RSV co-positivity rates were reported during the prevalence of Delta variant of concern (VOC) in comparison to Omicron VOC and pre-Delta VOC (2020–2022, US, children aged 0–18 years [39]), which highlights the need for any analysis on the SARS-CoV-2 incidence or clinical manifestations to be linked to a more precise context on the dominant SARS-CoV-2 lineages in the region.

The dominant SARS-CoV-2 PANGO lineages in Latvia during the study period were BA.5 and its sublineages, with the incidence maximum in September and October (exceeding 80% of the sequenced specimens per week) slowly and evenly declining since November until sporadically detected in spring 2023 (NMRL data). The incidences of SARS-CoV-2 from November to January, including the peak of the influenza epidemic, were shaped by a diverse mix of the sublineages, and the most frequently detected were BF (mostly BF.7), BN (mostly BN.1), and BQ.1, covering up to half of the sequenced specimens per week until the sublineages of XBB (mostly XBB.1) slowly replaced the landscape and dominated during the spring and summer 2023 (NMRL data). In parallel with the analysis by the multiplex real-time RT-qPCR methods, the SARS-CoV-2-positive respiratory specimens were randomly selected for variant detection by the whole genome sequencing (the workflow principles are described previously [40]). The PANGO lineage was detected for six SARS-CoV-2-positive specimens of this study set: BA.5.2.1 (also positive for RV, Week 48, age group 65<), CL.1 (PiV4, Week 49, age group 15–64), XBB.2 (IAV and EV/RV undifferentiated, Week 52, age group 65<), BQ.1.2 (CoV-OC43 and RV, Week 04, age group 5–14), EF.1 (RV, Week 06, age group 65<), BQ.1.1 (RV, Week 12, age group 65<), XBB.1 (RV, Week 16, age group 15–64) (NMRL data). Additional two SARS-CoV-2 respiratory specimens may enrich the observations. Those were collected from the patients (age group 65<) with the SARS-CoV-2-positive codetections a day before the diagnostic by the multiplex real-time RT-qPCR methods and identified as the representatives of the lineage BQ.1.1 (also positive for RSV B, Week 49) and BA.5.2.1 (IAV, Week 02) (NMRL data). The SARS-CoV-2 lineage spectrum of a few dozen of the SARS-CoV-2-mono-positive specimens resembled the regional picture. The obtained case count overlapping with our dataset was rather modest and limits the conclusions; nevertheless, the data on the SARS-CoV-2 sublineages add to the long-term observations and understanding of the respiratory virus co-circulation dynamic.

The assessment and comparison of any observations regarding virus-virus interactions ask for an advancement of the complementary computational methods within the knowledge of the seasonal and geographical as well as populational or timescale differences [1,16,17,41]. Our study focuses on the codetections of at least two virus groups per single specimen instead of a clinical representation of the caused illness. The specimens were collected from the patients with symptoms of a respiratory illness, and the study does not involve any data on the longevity and severity of the symptoms before and after the testing of the specimen. At an observational and general level, our results on a one-year scenery in Latvia agree with some conclusions from other regions, including emerging reports on the respiratory virus co-circulation early after the receding of the global SARS-CoV-2 incidence (Germany, 2022 [4]; Saudi Arabia, 2022–2023 [42]; and Turkey, 2021–2023 [43]). We compared the median age of the patients with the positive and multi-positive specimens among nine virus groups with a prevalence exceeding four percent of the positive cases per year. The majority of the multi-positive specimens (225/348) were contributed by the age group 0–4 (Table 1 and Table A1); nevertheless, the difference matrices for the children with multi-positive specimens (Figure 3E–G) resembled quite distantly the matrices for the complete multi-positive dataset (all age groups, Figure 3A–C). For example, the comparison highlighted the role of RSV and MPV in multi-positive specimens collected from the youngest children within the age group 0–4 (Table 3 and Figure 3), thus partially supporting observations elsewhere [18,23,44], whereas the pronounced trend of the MPV and RSV-multi-positives within the difference matrices for the small children aged 0–4 (Figure 3E,G) was less visible within the whole dataset of all age groups (Figure 3A–C). Our results illustrate the advantage of the summarised dataset, which provides an incidence overview among the entire age spectrum of the patients, as well as the results point out the need for separate analysis of the data on patients’ age among the dominant age groups, especially the young children (age group 0–4). Thus, the separated comparison within the overall dataset and the age group 0–4 provided a combined observation on the SARS-CoV-2 as a more pronounced burden for elderly patients as well as for the younger patients within the age group 0–4 (Table 3 and Figure 3). The results suggest a similar but less clear tendency for the RSV-positives (Table 3 and Figure 3). Besides these observations, the data on the patients’ age in our study linked some data on the seasonal incidence with an insight into a possible age lag between the RV-positive patients during the onset of the influenza epidemic (Table 3). Also, on the other hand, the data on the patients’ age limited some unreasonable analysis. Namely, the incidence of the codetections and mono-positives among the specimens sent from the regional hospitals was twice as high as obtained in the central hospitals or by sentinel practitioners (*p* < 0.001, Fisher’s exact test), which turned out to be consequent to the age bias of the patients among the institutions, i.e., the median age of the patients from the regional hospitals was 6 years (IQR 1–50.25), significantly different from 57 and 32 years (IQR 32.00–74.00 and 8.00–52.75, central hospitals and sentinel; *p* < 0.001, Mann–Whitney). Thus, any comparisons of the respiratory virus spread among the regions were unsuitable for our dataset. 

Our study illustrates the common limitations and difficulties in comparing any data on the respiratory virus circulation obtained by various methods. Implementing the multiplex real-time RT-qPCR methods into routine diagnostics makes it easier to acquire comparable and interpretable results. Nevertheless, the target composition of the assays raises questions on compatibility in terms of the versatility and coverage per virus group of immediate concern and retrospective interest in a long-term, regional, or cross-border manner. Combining the results enhances the limited choice between a possible data diversity loss or an exaggerated complexity for creating an optimally inclusive and summarised dataset. In the cases when differentiation per virus is not feasible for the whole dataset, the dilemma of deciding between the “undifferentiated” or “summarised” virus groups will remain a grey area for cautious interpretations despite any reasoning, and, moreover, any approach needs to be reconsidered each season regarding the actual viral occurrence in any region and subsequent analytical adjustments at the laboratory level. This retrospective study juggled the heterogeneity of the asymmetrical dataset, which spans one year only but also combines three testing approaches adapting to the diagnostic reality of the laboratory. 

In our case, the dataset provided a challenge to interpret the results obtained for EV, RV, and EV/RV-undifferentiated. The positive results for “EV” in our study, as provided by the method, would describe the specimens as positive for human coxsackievirus (seven types), human echovirus (eight types), and two EV types. The incomplete coverage of the specimens in terms of targeting the presence of EV limits any conclusions; nevertheless, the high incidence in terms of the codetections (72%, 23/32) during the pre-epidemic and the beginning–peak weeks of the epidemic (September–January) draws a question on the actual EV role in the respiratory virus co-circulation during the entire year in the periods when the specimens were not tested for the EV. Tactical exclusion of the EV-positives from the dataset would not affect the differences in the all-age and intra-age group positivity (Table A2) between the epidemic phases, but the exclusion of the EV data would reduce the overall (all age groups) incidence of the codetections. As a consequence, the observed difference in the codetection prevalence (Table A2) between the pre-epidemic and the epidemic would be lost (*p* > 0.05). Still, the difference between the pre- and post-epidemic would retain (relative risk 1.51, 95%CI 1.03 ÷ 2.23, *p* < 0.05, Fisher’s exact test), thus demonstrating the possible EV role in the observed overall positivity of the specimens per autumn. We chose not to exclude the results for EV detections from the dataset and interpreted any EV context accordingly. Such separate calculations for EV, RV, and EV/RV groups have already been implemented in the systematic retrospective studies of the accumulated database data or the literature [23,27]. 

Also, a multitude of studies on the respiratory virus co-circulation and monitoring had been reported, based on the methods targeting multiple viruses at once but non-distinguishing the picornavirus group [4,5,25,31,34,39,42,43,44,45] including the stressed need to add more precise follow-up analysis to separate between EV and RV, and subtyping [44,46]. Thus, we could have summarised the results for EV and RV-positives as EV/RV together with EV/RV-undifferentiated and achieved full coverage of the specimens tested for the picornavirus group. Then, in a summarised EV/RV approach, the results obtained for EV would blend into RV dominance, resulting in EV/RV-positivity of 12.6% (605/4814), and the prevalence of the codetections among the EV/RV-positives would be 32% (195/605), i.e., both percentages would be close (*p* > 0.05, Fisher’s exact test) to the results of RV detected without EV (Table 2). The same blend-in tendency would be observed if we deepened the comparison between age groups and the epidemic phases (Table A4). Noteworthy, the observed prevalence of the EV or RV-positive codetections among respective virus-positives (all age groups) during the pre-epidemic would drop significantly from 69% and 48% to 34% (*p* < 0.001 and *p* < 0.05, Fisher’s exact test) due to merging with the EV/RV-undifferentiated detections for the specimens collected from the adult patients (see Methods; Table A4). We may conclude that in the case of summarising the EV, RV, and EV/RV data, the actual prevalence of the EV-codetections among the EV-positives (Table 2 and Table A4) would become unnoticeable. Thus, this study illustrates the importance of the panel selection per age group of diagnostic interest. Moreover, a glance into the archive data from the previous seasons since December 2018 obtained by the multiplex panels enabling the differentiated detection of EV and RV (NMRL data) has let us observe the lasting prevalence of the codetections among EV-positives as a half (52%) during 2018–2019, followed by 38%, zero, and 35% for the next three seasons (without any data on SARS-CoV-2 among those specimens), jumping to 72% as observed in this study during five months since September 2022 (Table 2). The prevalence of the codetections among RV-positives in the previous years was 31%, 18%, 9%, and 35%, respectively, followed by 36% in 2022–2023 (Table 2). Summarising the EV and RV data into EV/RV yielded a blended prevalence of the codetections into 33%, 17%, 9%, and 32% per respective years, as could be calculated for 2022–2023. Therefore, our study emphasises the need to understand the actual impact of the EV on the common knowledge of the picornavirus role among other respiratory viruses.

Worldwide debate on the shift versus stabilisation of the respiratory virus circulation patterns is limited by the wait for the unfolding of the upcoming seasons to observe and model and to adjust the fit of the earlier models to understand the global, regional, and host-modulated trends to explain respiratory virus co-circulation in a new normality with SARS-CoV-2 as the latest member of the clinical interest. The asymmetrical availability of the diagnostical precision provides gaps in diagnostic coverage, highlighting the importance of the cautious interpretations regarding any available data and the context of some hidden co-circulation dynamic among the sublineages of any respiratory virus per respective season(s). The principal targets of the global surveillance interest are influenza viruses and SARS-CoV-2, followed by RSV. The routine use of the multiplex real-time RT-qPCR methods adds layered data on the other surrounding respiratory viruses. Any disproportional observations or incompatible differences in the results of the local and global scale could be explained as the consequences of the different methodological approaches together with the data collection regarding the age group representation.

## 5. Conclusions

The extended testing of the respiratory virus incidence within and along the frame of the respiratory virus monitoring programme provided an insight into the circulation and the multitude of the respiratory virus interactions in Latvia.

The data obtained for EV separately from RV highlighted the prevalence of the codetections among the EV-positives.

## Figures and Tables

**Table 1:** The number and the female:male (F:M) ratio of the specimens tested, and the number and the relative number of positives for at least one virus group, the number of multi- and mono-detections per age group of the patients during 1 September 2022–31 August 2023. The median age and interquartile range (IQR, measured in years) of the patients’ age groups regarding the multi- and mono-detections according to the influenza epidemic timeframe in Latvia. Abbreviations: “-” insufficient case count.

Timeframe	Age Group	ALL Specimens			POSITIVE Specimens			MEDIAN AGE (IQR)	
		Number Tested	F:M Ratio	Positives	Positives, %	Multi- Detections	Mono- Detections	Patients with Multi-Detections	Patients with Mono-Detections
year (1 September 2022–31 August 2023)									
	age 0–4	637	0.77 (277/360)	521	82	225	296	1.00 (1.00–2.00)	1.00 (0–3.00)
	age 5–14	355	0.87 (165/190)	233	66	56	177	7.50 (6.00–9.75)	7.00 (6.00–10.00)
	age 15–64	2062	0.98 (1021/1041)	646	31	43	603	36.00 (31.00–53.00)	42.00 (31.00–55.00)
	age 65<	1760	1.34 (1009/751)	449	26	24	425	77.00 (70.00–82.75)	77.00 (71.00–84.00)
	all specimens	4814	1.06 (2472/2342)	1849	38	348	1501	3.00 (1.00–8.75)	40.00 (7.00–67.00)
pre-epidemic (1 September–7 December 2022)									
	age 0–4	191	0.59 (71/120)	159	83	69	90	2.00 (0.00–3.00)	1.00 (0–3.00)
	age 5–14	58	1 (29/29)	35	60	11	24	6.00 (5.00–9.00)	7.50 (6.00–12.00)
	age 15–64	324	1.27 (181/143)	121	37	7	114	32.00 (22.00–62.00)	39.50 (27.00–52.00)
	age 65<	214	1.23 (118/96)	65	30	4	61	73.50 (68.00–80.50)	75 (70.00–82.00)
	all specimens	787	1.03 (399/388)	380	48	91	289	2.00 (1.00–4.00)	28.00 (3.00–59.50)
epidemic (2 December 2022–20 April 2023)									
	age 0–4	390	0.85 (179/211)	320	82	137	183	1.00 (1.00–2.00)	1.00 (0–3.00)
	age 5–14	268	0.87 (125/143)	178	66	41	137	8.00 (6.00–10.00)	7.00 (6.00–9.50)
	age 15–64	1278	0.92 (612/666)	428	33	31	397	34.00 (31.00–50.00)	44.00 (32.00–56.00)
	age 65<	1250	1.39 (726/524)	340	27	19	321	78.00 (70.00–86.00)	77.00 (71.00–84.00)
	all specimens	3186	1.06 (1642/1544)	1266	40	228	1038	3.00 (1.00–11.00)	44.00 (7.00–69.00)
post-epidemic (21 April–31 August 2023)									
	age 0–4	56	0.93 (27/29)	42	75	19	23	1.00 (1.00–3.00)	1.00 (0–2.00)
	age 5–14	29	0.61 (11/18)	20	69	4	16	8.00 (5.50–9.75)	7.00 (5.25–7.75)
	age 15–64	460	0.98 (228/232)	97	21	5	92	57.00 (35.50–60.00)	38.00 (28–51.75)
	age 65<	296	1.26 (165/131)	44	15	1	43	-	78.00 (71.00–84.00)
	all specimens	841	1.05 (431/410)	203	24	29	174	3.00 (1.00–9.50)	39 (18.75–64.25)

**Table 2:** The number of the specimens tested for the virus group, the number and the relative number of positives, multi- and mono-detections, the number of the specimens with two, three, four, and five codetections as well as F:M ratio of patients with positive specimens during 1 September 2022–31 August 2023. The total sum of positives exceeds the number of the positive specimens since each incidence is recounted per virus involved. Enterovirus (EV) tested until 31 January 2023; EV/RV undifferentiated picornavirus group not tested for the age group 0–4.

Virus Group	Tested	Positives	Positives, %	Multi- Detections	Mono- Detections	2–3–4–5 Detections per Specimen	F:M Ratio
Adenovirus, AdV	4814	274	5.69	151	123	111–33–5–2	0.94 (133/141)
Bocavirus, BoV	4748	157	3.31	123	34	79–38–4–2	0.65 (62/95)
Coronavirus 229E, CoV-229E	4748	13	0.27	5	8	2–2–1–0	0.63 (5/8)
Coronavirus NL63, CoV-NL63	4748	60	1.26	16	44	13–3–0–0	1.14 (32/28)
Coronavirus OC43, CoV-OC43	4748	85	1.79	36	49	25–11–0–0	0.89 (40/45)
Enterovirus, EV	1752	32	1.83	23	9	14–8–0–1	0.39 (9/23)
EV/RV undifferentiated	670	76	11.34	7	69	6–1–0–0	1.24 (42/34)
Influenza A virus, IAV	4814	322	6.69	51	271	40–11–0–0	1.08 (167/155)
Influenza B virus, IVB	4814	41	0.85	1	40	1–0–0–0	0.86 (19/22)
Metapneumovirus, MPV	4814	105	2.18	27	78	19–6–1–1	1.76 (67/38)
Parainfluenza virus, PiV (sum)	4814	88	1.83	39	49	29–7–2–1	0.76 (38/50)
PiV undifferentiated	2392	36	1.46	11	23	8–2–1–0	0.62 (13/21)
PiV1	2422	15	0.62	9	6	8–1–0–0	0.5 (5/10)
PiV2	2422	7	0.40	3	4	2–1–0–0	0.75 (3/4)
PiV3	2422	11	0.63	5	6	3–1–0–1	0.57 (4/7)
PiV4	2422	19	0.78	11	8	8–2–1–0	1.71 (12/7)
Respiratory syncytial virus, RSV (sum)	4814	259	5.38	72	187	58–12–2–0	0.89 (122/137)
RSV undifferentiated	3062	65	2.12	6	59	6–0–0–0	1.32 (37/28)
RSV A	1752	15	0.86	8	7	7–1–0–0	1.5 (9/6)
RSV B	1752	182	10.39	60	122	47–11–2–0	0.73 (77/105)
Rhinovirus, RV	4144	511	12.33	186	325	143–36–5–2	0.94 (248/263)
Severe acute respiratory syndrome coronavirus 2, SARS-CoV-2	4814	250	5.19	35	215	22–12–0–1	1.05 (128/122)

**Table 3:** The median age and interquartile range (IQR) of the patients whose specimens tested positive for at least one target (unspecified and per virus group) during the year of 1 September 2022–31 August 2023 and the epidemic periods (pre-epidemic, epidemic, and post-epidemic). The median age was measured in years for all patients and in months for the age group 0–4 years. The median age for patients with multi- and mono-positive specimens shown separately and evaluated by nonparametric Mann–Whitney U test; abbreviations of *p* values: * *p* < 0.05; ** *p* < 0.001; *** *p* < 0.001; **** *p* < 0.0001; N *p* > 0.05; “-” insufficient case count. EV tested until 31 January 2023; EV/RV not tested for the age group 0–4.

Virus Group	Timeframe	All Age Groups				Age Group 0–4 Years			
		Positives	Multi-Detections	Mono-Detections	*p*	Positives	Multi-Detections	Mono-Detections	*p*
unspecified	year	31.00 (4.0–64.0)	3.00 (1.0–8.8)	40.00 (7.0–67.0)	****	21.00 (8.5–37.0)	23.00 (12.0–35.0)	20.00 (6.0–37.8)	*
	pre-	12.00 (2.0–52.0)	2.00 (1.0–4.0)	28.00 (3.0–59.5)	****	22.00 (7.0–40.0)	26.00 (9.5–40.0)	20.50 (5.8–40.0)	N
	epid.	34.50 (4.0–67.0)	3.00 (1.0–11.0)	44.00 (7.0–69.0)	****	21.00 (9.0–37.0)	22.00 (12.0–35.0)	20.00 (5.0–38.0)	N
	post-	35.00 (7.0–62.0)	3.00 (1.0–9.5)	39.00 (18.8–64.3)	****	22.50 (12.5–33.0)	26.00 (9.5–40.0)	20.50 (5.8–40.0)	N
AdV	year	3.00 (2.0–8.0)	2.00 (1.0–5.0)	6.00 (2.0–30.0)	****	29.00 (19.0–42.0)	29.00 (17.0–40.0)	29.00 (21.0–43.0)	N
	pre-	3.00 (2.0–5.0)	3.00 (2.0–4.0)	3.00 (2.3–7.0)	N	32.50 (23.5–45.0)	30.00 (20.0–46.0)	40.00 (27.0–44.0)	N
	epid.	3.00 (1.0–8.5)	2.00 (1.0–5.0)	6.00 (2.0–28.5)	****	27.50 (16.0–38.8)	27.00 (14.5–38.0)	29.00 (19.0–43.0)	N
	post-	5.00 (2.0–31.5)	2.50 (1.0–6.5)	13.50 (3.5–38.3)	*	28.50 (20.3–42.8)	30.00 (21.0–46.0)	28.00 (15.5–41.5)	N
BoV	year	2.00 (1.0–7.0)	2.00 (1.0–4.0)	7.00 (1.0–45.8)	**	22.00 (12.0–34.0)	22.00 (14.8–34.3)	18.00 (8.5–22.0)	N
	pre-	2.00 (1.0–3.0)	2.00 (0.5–3.0)	1.00 (0.8–13.0)	N	22.00 (10.3–34.5)	24.00 (7.0–35.0)	22.00 (13.0–40.0)	N
	epid.	2.00 (1.0–8.0)	2.00 (1.0–5.0)	12.50 (1.0–47.3)	**	21.50 (12.0–34.0)	22.00 (14.0–34.0)	11.00 (6.0–21.0)	*
	post-	3.00 (1.0–58.0)	1.00 (1.0–43.0)	47.50 (10.0–61.8)	N	22.00 (21.5–34.5)	22.00 (21.3–40.3)	22.00 (22.0–22.0)	-
CoV-229E	year	37.00 (8.5–62.0)	10.00 (5.5–32.5)	52.00 (37.5–73.5)	*	-	-	-	-
	pre-	-	-	-	-	-	-	-	-
	epid.	35.00 (9.3–57.0)	10.00 (7.0–37.0)	52.00 (33.0–72.0)	N	-	-	-	-
	post-	51.50 (22.0–74.0)	-	63.00 (51.3–74.0)	-	-	-	-	-
CoV-NL63	year	35.50 (5.0–60.5)	4.50 (3.3–37.8)	40.00 (22.0–68.0)	**	22.00 (19.0–58.5)	50.00 (21.3–59.0)	19.00 (4.0–32.5)	*
	pre-	-	-	-	-	-	-	-	-
	epid.	41.00 (10.3–65.0)	8.00 (3.5–41.0)	49.00 (22.0–72.0)	*	31.50 (11.3–55.3)	50.50 (26.3–59.0)	14.50 (2.0–38.3)	N
	post-	24.00 (4.3–54.0)	4.00 (1.0–28.0)	28.00 (13.5–62.0)	N	-	-	-	-
CoV-OC43	year	28.00 (4.0–59.0)	6.50 (1.3–31.8)	49.00 (20.0–67.0)	****	22.00 (11.0–40.5)	22.00 (13.0–40.0)	24.50 (2.8–46.8)	N
	pre-	-	4.00 (4.0–4.0)	54.00 (54.0–54.0)	-	-	-	-	-
	epid.	25.00 (3.0–56.8)	4.50 (1.0–28.0)	36.50 (13.5–64.3)	****	22.00 (11.0–40.0)	22.00 (15.0–39.0)	24.50 (2.8–46.8)	N
	post-	63.00 (35.5–70.5)	43.00 (14.0–65.5)	67.00 (64.0–76.8)	-	22.00 (19.0–58.5)	-	-	-
EV									
pre-	year	44.50 (29.5–62.0)	62.00 (32.0–68.0)	43.00 (28.5–57.5)	N	-	-	-	-
	pre-	42.00 (28.0–62.0)	62.00 (27.0–65.5)	42.00 (27.8–55.5)	N	-	-	-	-
	epid.	48.00 (35.0–70.0)	-	-	-	-	-	-	-
	post-	44.00 (26.5–62.0)	-	44.00 (26.5–62.0)	-	-	-	-	-
IAV	year	34.50 (7.0–63.0)	7.00 (2.0–50.0)	40.00 (8.0–64.0)	****	30.00 (16.0–47.0)	26.00 (16.0–39.5)	38.00 (14.8–47.0)	N
	pre-	39.00 (25.0–62.5)	24.00 (0–51.0)	47.50 (29.0–65.8)	N	4.00 (4.0–4.0)	-	-	-
	epid.	33.50 (6.3–63.0)	7.00 (2.0–46.0)	39.00 (7.3–64.0)	****	30.00 (16.0–47.0)	27.00 (16.3–41.8)	38.00 (14.8–47.0)	N
	post-	-	-	-	-	-	-	-	-
IVB	year	32.00 (18.5–37.5)	-	32.00 (19.5–37.8)	-	4.00 (4.0–52.0)	-	-	-
	pre-	-	-	-	-	-	-	-	-
	epid.	32.00 (18.0–37.0)	-	32.50 (20.3–37.3)	-	-	-	-	-
	post-	29.00 (18.8–41.3)	-	29.00 (18.8–41.3)	-	-	-	-	-
MPV	year	38.00 (2.0–66.0)	2.00 (0–6.0)	51.50 (22.3–73.3)	****	12.00 (5.0–35.0)	12.00 (4.0–35.0)	12.00 (5.3–43.0)	N
	pre-	50.00 (8.0–72.0)	2.00 (1.0–6.0)	59.00 (32.0–74.3)	***	35.00 (21.0–53.0)	32.00 (11.0–48.5)	40.00 (21.0–56.0)	N
	epid.	34.00 (1.0–60.0)	1.50 (0–6.3)	51.00 (4.0–71.0)	****	7.00 (4.3–29.0)	7.00 (4.0–33.0)	7.00 (5.0–29.5)	N
	post-	40.00 (33.0–88.0)	-	40.00 (33.0–88.0)	-	-	-	-	-
PiV	year	6.00 (1.3–54.0)	2.00 (1.0–4.0)	42.00 (6.0–70.5)	****	22.00 (14.0–34.0)	22.00 (15.8–35.0)	23.00 (1.0–33.0)	N
	pre-	3.00 (1.0–12.5)	2.00 (1.0–4.0)	8.00 (2.5–53.0)	**	23.00 (8.0–35.5)	23.00 (10.0–35.0)	25.50 (1.8–40.8)	N
	epid.	31.50 (1.3–65.0)	2.00 (1.0–32.5)	58.50 (25.5–76.0)	**	20.00 (8.0–24.0)	20.00 (17.0–27.0)	0.50 (0–1.00)	-
	post-	26.50 (2.0–57.8)	2.00 (1.0–17.3)	37.00 (13.3–73.3)	*	30.00 (22.3–32.8)	30.00 (21.5–41.0)	30.00 (23.0–33.0)	N
RSV	year	19.00 (1.0–68.0)	2.00 (0–8.0)	47.00 (1.0–73.0)	****	11.00 (2.5–28.5)	17.50 (4.0–30.0)	7.00 (2.0–25.0)	N
	pre-	4.50 (1.0–64.5)	2.00 (0–5.0)	25.00 (2.0–73.0)	***	17.00 (4.0–32.0)	20.00 (5.8–36.3)	9.00 (2.0–31.0)	N
	epid.	47.50 (1.0–71.8)	2.00 (0–55.0)	59.50 (1.0–73.3)	*	6.00 (2.0–20.0)	9.50 (2.0–24)	4.00 (1.0–18.5)	N
	post-	-	-	-	-	-	-	-	-
RV	year	9.00 (2.0–54.0)	2.00 (1.0–6.3)	32.00 (6.0–62.5)	****	22.00 (10.0–35.0)	23.00 (12.0–35.8)	19.00 (5.0–34.0)	*
	pre-	3.00 (1.0–15.0)	2.00 (1.0–4.0)	12.00 (1.0–38.0)	***	22.00 (6.0–37.0)	26.00 (10.0–40.5)	10.00 (1.8–25.3)	*
	epid.	16.00 (3.0–60.0)	3.00 (1.0–11.5)	40.50 (7.0–67.3)	****	22.00 (12.0–35.3)	22.00 (12.0–35.0)	22.00 (9.3–37.8)	N
	post-	9.00 (2.0–43.0)	2.00 (1.0–4.5)	28.00 (5.3–62.0)	****	21.50 (5.5–32.8)	22.00 (12.5–41.5)	13.00 (4.0–31.0)	N
SARS-CoV-2	year	65.00 (40.8–78.0)	9.00 (2.0–70.0)	67.00 (48.0–78.0)	****	12.00 (4.0–28.5)	22.00 (4.0–38.0)	8.00 (2.5–18.0)	N
	pre-	44.50 (2.8–69.5)	62.00 (2.0–79.0)	42.00 (8.0–66.0)	N	16.00 (8.3–28.8)	32.00 (14.0–42.0)	9.00 (4.5–18.5)	-
	epid.	67.50 (43.8–79.0)	8.50 (1.8–70.8)	69.00 (52.3–79.8)	****	11.50 (3.3–28.8)	22.00 (4.0–38.0)	7.00 (2.0–16.0)	N
	post-	60.50 (44.0–75.0)	29.50 (0–59.0)	61.50 (45.5–77.0)	-	-	-	-	-

## Data Availability

The data presented in the study are included in the article. Further inquiries can be directed to the corresponding author as long as they meet the terms of the Institutional Review Board statement.

## Supplementary Materials

The following supporting information can be downloaded at: https://www.mdpi.com/article/10.3390/v16111650/s1, Figure S1: The number of the specimens positive for adenovirus (AdV), bocavirus (BoV), coronaviruses (CoV-229E, CoV-NL63, and CoV-OC43, enterovirus (EV), undifferentiated picornavirus group (EV/RV), influenza A virus (IAV), influenza B virus (IVB), metapneumovirus (MPV), parainfluenza virus 1/2/3/4 (PiV), respiratory syncytial virus A/B (RSV), rhinovirus (RV), and severe acute respiratory syndrome coronavirus 2 (SARS-CoV-2) per each week from 1 September 2022 to 31 August 2023; period of influenza epidemic (8 December 2022–20 April 2023) marked in blue. Abbreviations: * EV tested until 31 January 2023; ** EV/RV not tested for the age group 0–4.

## Appendix A

**Table A1:** The difference in the proportions of the positives and the specimens with the multi-detections between the age groups of the patients during the year (1 September 2022–31 August 2023) and the influenza epidemic periods in terms of the relative risk value with 95% confidence interval and Fisher’s exact test *p* value (* *p* < 0.05; ** *p* < 0.001; *** *p* < 0.001; **** *p* < 0.0001; N *p* > 0.05).

	Age Groups Compared	Year (1 September 2022–31 August 2023)	Pre-Epidemic (1 September–7 December 2022)	Epidemic (8 December 2022–20 April 2023)	Post-Epidemic (21 April–31 August 2023)
the difference in the proportion of the positives					
	0–4 vs. 5–14	1.246 (1.150 ÷ 1.360) ****	1.380 (1.141 ÷ 1.765) ***	1.235 (1.126 ÷ 1.368) ****	N
	0–4 vs. 15–64	2.611 (2.425 ÷ 2.810) ****	2.229 (1.915 ÷ 2.611) ****	2.450 (2.238 ÷ 2.681) ****	3.557 (2.776 ÷ 4.447) ****
	0–4 vs. 65<	3.206 (2.937 ÷ 3.502) ****	2.741 (2.233 ÷ 3.412) ****	3.017 (2.725 ÷ 3.341) ****	5.045 (3.677 ÷ 6.873) ****
	5–14 vs. 15–64	2.095 (1.893 ÷ 2.306) ****	1.616 (1.229 ÷ 2.041) **	1.983 (1.762 ÷ 2.218) ****	3.271 (2.312 ÷ 4.275) ****
	5–14 vs. 65<	2.573 (2.300 ÷ 2.866) ****	1.987 (1.464 ÷ 2.630) ****	2.442 (2.150 ÷ 2.759) ****	4.639 (3.120 ÷ 6.557) ****
	15–64 vs. 65<	1.228 (1.109 ÷ 1.361) ****	N	1.231 (1.093 ÷ 1.387) ***	1.419 (1.030 ÷ 1.967) *
	grouped 0–14 vs. 15<	2.653 (2.495 ÷ 2.819) ****	2.254 (1.973 ÷ 2.579) ****	2.491 (2.314 ÷ 2.679) ****	3.911 (3.179 ÷ 4.731) ****
the difference in the proportion of the multi-detections					
	0–4 vs. 5–14	1.797 (1.411 ÷ 2.316) ****	N	1.859 (1.394 ÷ 2.516) ****	N
	0–4 vs. 15–64	6.488 (4.799 ÷ 8.810) ****	7.501 (3.698 ÷ 15.63) ****	5.911 (4.138 ÷ 8.496) ****	8.776 (3.656 ÷ 21.46) ****
	0–4 vs. 65<	8.079 (5.446 ÷ 12.08) ****	7.052 (2.881 ÷ 18.20) ****	7.661 (4.906 ÷ 12.07) ****	19.90 (3.706 ÷ 114.7) ****
	5–14 vs. 15–64	3.611 (2.500 ÷ 5.201) ****	5.433 (2.314 ÷ 12.60) ***	3.180 (2.067 ÷ 4.880) ****	3.880 (1.182 ÷ 12.05) *
	5–14 vs. 65<	4.496 (2.877 ÷ 7.041) ****	5.107 (1.853 ÷ 14.32) **	4.122 (2.484 ÷ 6.854) ****	8.800 (1.402 ÷ 56.39) *
	15–64 vs. 65<	N	N	N	N
	grouped 0–14 vs. 15<	6.091 (4.753 ÷ 7.823) ****	6.973 (3.910 ÷ 12.65) ****	5.490 (4.107 ÷ 7.360) ****	8.718 (3.852 ÷ 19.97) ****

## Appendix B

**Table A2:** The difference in the proportions of the positives and the specimens with the multi-detections for each age group of the patients between three influenza epidemic periods of the year (1 September 2022–31 August 2023) in terms of the relative risk value with 95% confidence interval and Fisher’s exact test *p* value (* *p* < 0.05; ** *p* < 0.001; **** *p* < 0.0001; N *p* > 0.05).

Timeframe	Age Group	The Difference in the Proportion of Positives	The Difference in the Proportion of the Multi-Detections
pre-epidemic vs. epidemic			
	0–4	N	N
	5–14	N	N
	15–64	N	N
	65<	N	N
	all age	1.215 (1.115 ÷ 1.319) ****	1.330 (1.070 ÷ 1.642) *
	grouped 0–14	N	N
	grouped 15<	N	N
epidemic vs. post-epidemic			
	0–4	N	N
	5–14	N	N
	15–64	1.588 (1.314 ÷ 1.932) ****	N
	65<	1.830 (1.383 ÷ 2.449) ****	N
	all age	1.646 (1.453 ÷ 1.873) ****	N
	grouped 0–14	N	N
	grouped 15<	1.629 (1.391 ÷ 1.915) ****	N
pre-epidemic vs. post-epidemic			
	0–4	N	N
	5–14	N	N
	15–64	1.771 (1.413 ÷ 2.220) ****	N
	65<	2.043 (1.458 ÷ 2.869) ****	N
	all age	2.000 (1.741 ÷ 2.303) ****	1.676 (1.154 ÷ 2.463) **
	grouped 0–14	N	N
	grouped 15<	1.854 (1.535 ÷ 2.239) ****	N

## Appendix C

**Table A3:** The number of the specimens tested and the number of positives for at least one respiratory virus group, multi- and mono-detections per age group and sex of the patients during 1 September 2022–31 August 2023 according to the timeframe of the influenza epidemic in Latvia. The difference in the proportions of the positives and the specimens with the multi-detections between the sex of the patients within each age group and timeframe of the epidemic in terms of the relative risk value with 95% confidence interval and Fisher’s exact test *p* value (N *p* > 0.05).

Timeframe	Age Group	Sex	Tested	Positives	Multi-Detections	Mono-Detections	The Difference in the Proportion of Positives	The Difference in the Proportion of Multi-Positives
year (1 September 2022–31 August 2023)								
	0–4	F	277	230	97	133	N	N
		M	360	291	128	163		
	5–14	F	165	112	26	86	N	N
		M	190	121	30	91		
	15–64	F	1021	337	24	313	N	N
		M	1041	309	19	290		
	65<	F	1009	243	14	229	N	N
		M	751	206	10	196		
	all age	F	2472	922	161	761	N	N
		M	2342	927	187	740		
pre-epidemic (1 September–7 December 2022)								
	0–4	F	71	58	28	30	N	N
		M	120	101	41	60		
	5–14	F	29	18	4	14	N	N
		M	29	17	7	10		
	15–64	F	181	68	3	65	N	N
		M	143	53	4	49		
	65<	F	118	37	1	36	N	N
		M	96	28	3	25		
	all age	F	399	181	36	145	N	N
		M	388	199	55	144		
epidemic (2 December 2022–20 April 2023)								
	0–4	F	179	150	59	91	N	N
		M	211	170	78	92		
	5–14	F	125	86	19	67	N	N
		M	143	92	22	70		
	15–64	F	612	216	20	196	N	N
		M	666	212	11	201		
	65<	F	726	184	13	171	N	N
		M	524	156	6	150		
	all age	F	1642	636	111	525	N	N
		M	1544	630	117	513		
post-epidemic (21 April–31 August 2023)								
	0–4	F	27	22	10	12	N	N
		M	29	20	9	11		
	5–14	F	11	8	3	5	N	N
		M	18	12	1	11		
	15–64	F	228	53	1	52	N	N
		M	232	44	4	40		
	65<	F	165	22	0	22	N	N
		M	131	22	1	21		
	all age	F	431	105	14	91	N	N
		M	410	98	15	83		

## Appendix D

**Table A4:** The number of the specimens tested for the respiratory virus group and the number of positives, multi- and mono-detections, as well as female:male (F:M) ratio of patients with positive specimens per age group of the patients during 1 September 2022–31 August 2023, and the number of positives and multi-detections during the influenza epidemic periods. “N”—not applicable per category.

Virus Group	Age Group	Year					Pre-Epidemic		Epidemic		Post-Epidemic	
		Tested	Positives	Multi-Detections	Mono-Detections	F:M Ratio	Positives	Multi-Detections	Positives	Multi-Detections	Positives	Multi-Detections
Adenovirus, AdV												
	0–4	637	166	111	55	0.89 (78/88)	42	27	108	73	16	11
	5–14	355	59	31	28	1.11 (31/28)	11	6	40	21	8	4
	15–64	2062	40	7	33	1.00 (20/20)	5	1	26	5	9	1
	65<	1760	9	2	7	0.80 (4/5)	1	1	7	1	1	0
Bocavirus, BoV												
	0–4	622	107	94	13	0.67 (43/64)	20	15	78	71	9	8
	5–14	346	20	15	5	0.67 (8/12)	2	2	18	13	0	0
	15–64	2033	22	9	13	0.69 (9/13)	1	0	16	7	5	2
	65<	1747	8	5	3	0.33 (2/6)	0	0	7	4	1	1
Coronavirus 229E, CoV-229E												
	0–4	622	1	1	0	N (0/1)	0	0	0	0	1	1
	5–14	346	3	2	1	2.00 (2/1)	1	0	2	2	0	0
	15–64	2033	6	2	4	1.00 (3/3)	0	0	3	1	3	1
	65<	1747	3	0	3	N (0/3)	0	0	1	0	2	0
Coronavirus NL63, CoV-NL63												
	0–4	622	13	8	5	0.63 (5/8)	0	0	8	4	5	4
	5–14	346	6	2	4	5.00 (5/1)	0	0	3	1	3	1
	15–64	2033	27	6	21	0.93 (13/14)	0	0	18	4	9	2
	65<	1747	14	0	14	1.80 (9/5)	0	0	11	0	3	0
Coronavirus OC43, CoV-OC43												
	0–4	622	25	17	8	1.08 (13/12)	1	1	23	15	1	1
	5–14	346	9	6	3	0.80 (4/5)	0	0	9	6	0	0
	15–64	2033	35	11	24	1.19 (19/16)	1	0	30	8	4	3
	65<	1747	16	2	14	0.33 (4/12)	0	0	12	1	4	1
Enterovirus, EV												
	0–4	375	29	21	8	0.32 (7/22)	24	17	5	4	N	N
	5–14	178	1	1	0	N (0/1)	1	1	0	0	N	N
	15–64	628	1	1	0	N (1/0)	0	0	1	1	N	N
	65<	571	1	0	1	N (1/0)	1	0	0	0	N	N
EV/RV undifferentiated												
	0–4	N	N	N	N	N	N	N	N	N	N	N
	5–14	2	N	N	N	N	N	N	0	0	N	N
	15–64	463	60	5	55	1.22 (33/27)	39	4	10	1	11	0
	65<	205	16	2	14	1.29 (9/7)	8	1	5	1	3	0
Influenza A virus, IAV												
	0–4	637	51	21	30	1.13 (27/24)	1	1	50	20	0	0
	5–14	355	74	13	61	0.95 (36/38)	0	0	74	13	0	0
	15–64	2062	128	11	117	1.03 (65/63)	10	2	117	9	1	0
	65<	1760	69	6	63	1.30 (39/30)	2	0	67	6	0	0
Influenza B virus, IVB												
	0–4	637	3	1	2	N (0/3)	0	0	1	1	2	0
	5–14	355	5	0	5	0.67 (2/3)	0	0	4	0	1	0
	15–64	2062	31	0	31	1.07 (16/15)	0	0	17	0	14	0
	65<	1760	2	0	2	1.00 (1/1)	0	0	1	0	1	0
Metapneumovirus, MPV												
	0–4	637	35	19	16	1.33 (20/15)	7	4	28	15	0	0
	5–14	355	7	5	2	0.40 (2/5)	1	1	6	4	0	0
	15–64	2062	36	3	33	1.77 (23/13)	13	0	21	3	2	0
	65<	1760	27	0	27	4.40 (22/5)	10	0	16	0	1	0
Parainfluenza virus, PiV (sum)												
	0–4	637	41	30	11	0.41 (12/29)	22	16	11	9	8	5
	5–14	355	8	3	5	1.00 (4/4)	6	2	2	1	0	0
	15–64	2062	23	4	19	0.92 (11/12)	5	1	10	2	8	1
	65<	1760	16	2	14	2.20 (11/5)	3	0	9	2	4	0
PiV undifferentiated												
	0–4	262	12	8	4	0.20 (2/10)	N	N	4	3	8	5
	5–14	175	1	0	1	N (0/1)	N	N	1	0	0	0
	15–64	971	12	2	10	0.71 (5/7)	N	N	4	1	8	1
	65<	984	9	1	8	2.00 (6/3)	N	N	5	1	4	0
PiV1												
	0–4	375	8	6	2	0.33 (2/6)	8	6	0	0	0	0
	5–14	180	3	2	1	0.50 (1/2)	3	2	0	0	0	0
	15–64	1091	3	1	2	0.50 (1/2)	3	1	0	0	0	0
	65<	776	1	0	1	N (1/0)	1	0	0	0	0	0
PiV2												
	0–4	375	4	3	1	0.33 (1/3)	3	2	1	1	0	0
	5–14	180	2	0	2	1.00 (1/1)	2	0	0	0	0	0
	15–64	1091	1	0	1	N (1/0)	0	0	1	0	0	0
	65<	776	0	0	0	N	0	0	0	0	0	0
PiV3												
	0–4	375	8	5	3	0.33 (2/6)	6	3	2	2	0	0
	5–14	180	0	0	0	N	0	0	0	0	0	0
	15–64	1091	2	0	2	1.00 (1/1)	1	0	1	0	0	0
	65<	776	1	0	1	N (1/0)	0	0	1	0	0	0
PiV4												
	0–4	375	9	8	1	1.25 (5/4)	5	5	4	3	0	0
	5–14	180	2	1	1	N (2/0)	1	0	1	1	0	0
	15–64	1091	4	1	3	1.00 (2/2)	1	0	3	1	0	0
	65<	776	4	1	3	3.00 (3/1)	2	0	2	1	0	0
Respiratory syncytial virus, RSV (sum)												
	0–4	637	113	48	65	0.61 (43/70)	61	30	52	18	0	0
	5–14	355	12	8	4	0.50 (4/8)	9	6	3	2	0	0
	15–64	2062	53	8	45	1.41 (31/22)	22	4	31	4	0	0
	65<	1760	81	8	73	1.19 (44/37)	30	2	50	6	1	0
RSV undifferentiated												
	0–4	262	4	1	3	1.00 (2/2)	N	N	4	1	0	0
	5–14	177	0	0	0	N	N	N	0	0	0	0
	15–64	1434	26	4	22	1.36 (15/11)	13	3	13	1	0	0
	65<	1189	35	1	34	1.33 (20/15)	14	1	20	0	1	0
RSV A												
	0–4	375	10	5	5	1.50 (6/4)	6	4	4	1	N	N
	5–14	178	2	2	0	1.00 (1/1)	0	0	2	2	N	N
	15–64	628	0	0	0	N	0	0	0	0	N	N
	65<	571	3	1	2	2.00 (2/1)	1	0	2	1	N	N
RSV B												
	0–4	375	101	44	57	0.55 (36/65)	57	28	44	16	N	N
	5–14	178	10	6	4	0.43 (3/7)	9	6	1	0	N	N
	15–64	628	27	4	23	1.45 (16/11)	9	1	18	3	N	N
	65<	571	44	6	38	1.00 (22/22)	15	1	29	5	N	N
Rhinovirus, RV												
	0–4	637	197	128	69	0.91 (94/103)	59	41	114	74	24	13
	5–14	353	83	29	54	0.77 (36/47)	15	5	56	20	12	4
	15–64	1599	146	16	130	1.06 (75/71)	21	1	106	15	19	0
	65<	1555	85	13	72	1.02 (43/42)	4	1	71	12	10	0
Severe acute respiratory syndrome coronavirus 2, SARS-CoV-2												
	0–4	637	29	15	14	0.53 (10/19)	8	3	20	11	1	1
	5–14	355	8	3	5	3.00 (6/2)	1	0	6	3	1	0
	15–64	2062	85	7	78	1.02 (43/42)	11	1	56	5	18	1
	65<	1760	128	10	118	1.17 (69/59)	10	3	104	7	14	0

## Appendix E

**Table A5:** The combinations of respiratory virus groups and the number of the specimens detected during 1 September 2022–31 August 2023 per combination, patients’ age groups and the epidemic periods. The summarised result for respiratory syncytial virus (RSV) and parainfluenza virus (PiV) shown also in the available form—as detailed subtyping or undifferentiated depending on the multiplex real-time RT-qPCR panel; enterovirus (EV) tested until 31 January 2023; EV/RV undifferentiated picornavirus group (age group 0–4 not tested). “N”—no detections per category. At-a-glance overview of the combination count per virus group shown in Figure 2.

Combinations		Virus Groups																					
Age Group	Year (Pre-Epidemic; Epidemic; Post-Epidemic)	AdV	BoV	CoV-229E	CoV-NL63	CoV-OC43	EV	EV/RV	IAV	IVB	MPV	PiV (sum)	PiV und.	PiV1	PiV2	PiV3	PiV4	RSV (sum)	RSV und.	RSV A	RSV B	RV	SARS-CoV-2
AdV, RV																							
0–4 5–14	29 (6; 20; 3) 10 (1; 6; 3)	39	-	-	-	-	-	-	-	-	-	-	-	-	-	-	-	-	-	-	-	39	-
BoV, RV																							
0–4 5–14 65<	20 (4; 14; 2) 5 (1; 4; N) 2 (N; 2; N)	-	27	-	-	-	-	-	-	-	-	-	-	-	-	-	-	-	-	-	-	27	-
RSV, RV																							
0–4 5–14 15–64 65<	16 (9; 7; N) 2 (2; N; N) 2 (1; 1; N) 4 (N; 4; N)	-	-	-	-	-	-	-	-	-	-	-	-	-	-	-	-	24	1	3	20	24	-
AdV, BoV																							
0–4 5–14 15–64	15 (1; 12; 2) 4 (1; 3; N) 1 (N; 1; N)	20	20	-	-	-	-	-	-	-	-	-	-	-	-	-	-	-	-	-	-	-	-
AdV, IAV																							
0–4 5–14 15–64	5 (N; 5; N) 6 (N; 6; N) 2 (1; 1; N)	13	-	-	-	-	-	-	13	-	-	-	-	-	-	-	-	-	-	-	-	-	-
AdV, RSV																							
0–4 5–14 15–64	10 (8; 2; N) 2 (1; 1; N) 1 (N; 1; N)	13	-	-	-	-	-	-	-	-	-	-	-	-	-	-	-	13	-	3	12	-	-
AdV, BoV, RV																							
0–4 5–14	10 (2; 7; 1) 1 (N; 1; N)	11	11	-	-	-	-	-	-	-	-	-	-	-	-	-	-	-	-	-	-	11	-
BoV, CoV-OC43																							
0–4 5–14 15–64 65<	2 (N; 2; N) 1 (N; 1; N) 6 (N; 5; 1) 2 (N; 1; 1)	-	11	-	-	11	-	-	-	-	-	-	-	-	-	-	-	-	-	-	-	-	-
BoV, IAV																							
0–4 5–14	7 (N; 7; N) 3 (N; 3; N)	-	10	-	-	-	-	-	10	-	-	-	-	-	-	-	-	-	-	-	-	-	-
CoV-OC43, RV																							
0–4 5–14 15–64	4 (N; 3; 1) 2 (N; 2; N) 3 (N; 3; N)	-	-	-	-	9	-	-	-	-	-	-	-	-	-	-	-	-	-	-	-	9	-
PiV, RV																							
0–4 5–14 65<	7 (3; 2; 2) 1 (N; 1; N) 1 (N; 1; N)	-	-	-	-	-	-	-	-	-	-	9	5	1	1	-	2	-	-	-	-	9	-
AdV, PiV																							
0–4 5–14 65<	5 (4; N; 1) 1 (1; N; N) 1 (N; 1; N)	7	-	-	-	-	-	-	-	-	-	7	1	4	1	-	1	-	-	-	-	-	-
CoV-NL63, RV																							
0–4 15–64	5 (N; 4; 1) 2 (N; 2; N)	-	-	-	7	-	-	-	-	-	-	-	-	-	-	-	-	-	-	-	-	7	-
EV, RV																							
0–4 15–64	6 (6; N; N) 1 (N; 1; N)	-	-	-	-	-	7	-	-	-	-	-	-	-	-	-	-	-	-	-	-	7	-
IAV, RSV																							
0–4 5–14 15–64 65<	1 (N; 1; N) 1 (N; 1; N) 3 (1; 2; N) 2 (N; 2; N)	-	-	-	-	-	-	-	7	-	-	-	-	-	-	-	-	7	1	1	5	-	-
MPV, RV																							
0–4 5–14 15–64	3 (1; 2; N) 2 (N; 2; N) 2 (N; 2; N)	-	-	-	-	-	-	-	-	-	7	-	-	-	-	-	-	-	-	-	-	7	-
RV, SARS-CoV-2																							
0–4 15–64 65<	1 (N; N; 1) 1 (N; 1; N) 5 (1; 4; N)	-	-	-	-	-	-	-	-	-	-	-	-	-	-	-	-	-	-	-	-	7	7
IAV, RV																							
0–4 5–14 15–64	2 (1; 1; N) 1 (N; 1; N) 3 (N; 3; N)	-	-	-	-	-	-	-	6	-	-	-	-	-	-	-	-	-	-	-	-	6	-
AdV, BoV, CoV-OC43																							
0–4	4 (N; 4; N)	4	4	-	-	4	-	-	-	-	-	-	-	-	-	-	-	-	-	-	-	-	-
AdV, CoV-NL63																							
0–4 5–14 15–64	2 (N; N; 2) 1 (N; 1; N) 1 (N; N; 1)	4	-	-	4	-	-	-	-	-	-	-	-	-	-	-	-	-	-	-	-	-	-
AdV, CoV-OC43																							
0–4 5–14	3 (N; 3; N) 1 (N; 1; N)	4	-	-	-	4	-	-	-	-	-	-	-	-	-	-	-	-	-	-	-	-	-
AdV, MPV																							
0–4 5–14	2 (N; 2; N) 2 (1; 1; N)	4	-	-	-	-	-	-	-	-	4	-	-	-	-	-	-	-	-	-	-	-	-
AdV, SARS-CoV-2																							
0–4 65<	3 (N; 3; N) 1 (1; N; N)	4	-	-	-	-	-	-	-	-	-	-	-	-	-	-	-	-	-	-	-	-	4
BoV, PiV																							
0–4	4 (1; 3; N)	-	4	-	-	-	-	-	-	-	-	4	1	1	-	1	1	-	-	-	-	-	-
IAV, SARS-CoV-2																							
15–64 65<	2 (N; 2; N) 2 (N; 2; N)	-	-	-	-	-	-	-	4	-	-	-	-	-	-	-	-	-	-	-	-	-	4
AdV, RV, SARS-CoV-2																							
0–4 15–64	2 (N; 2; N) 1 (N; 1; N)	3	-	-	-	-	-	-	-	-	-	-	-	-	-	-	-	-	-	-	-	3	3
BoV, IAV, RV																							
0–4 65<	2 (N; 2; N) 1 (N; 1; N)	-	3	-	-	-	-	-	3	-	-	-	-	-	-	-	-	-	-	-	-	3	-
BoV, MPV																							
0–4 5–14	2 (N; 2; N) 1 (N; 1; N)	-	3	-	-	-	-	-	-	-	3	-	-	-	-	-	-	-	-	-	-	-	-
EV, RSV																							
0–4 5–14	2 (N; 2; N) 1 (N; 1; N)	-	-	-	-	-	3	-	-	-	-	-	-	-	-	-	-	3	-	-	3	-	-
EV/RV, RSV																							
15–64 65<	2 (2; N; N) 1 (1; N; N)	-	-	-	-	-	-	3	-	-	-	-	-	-	-	-	-	3	3	-	-	-	-
AdV, BoV, IAV																							
0–4	2 (N; 2; N)	2	2	-	-	-	-	-	2	-	-	-	-	-	-	-	-	-	-	-	-	-	-
AdV, BoV, RSV																							
0–4	2 (N; 2; N)	2	2	-	-	-	-	-	-	-	-	-	-	-	-	-	-	2	-	-	2	-	-
AdV, EV																							
0–4	2 (1; 1; N)	2	-	-	-	-	2	-	-	-	-	-	-	-	-	-	-	-	-	-	-	-	-
AdV, RSV, RV																							
0–4 5–14	1 (1; N; N) 1 (1; N; N)	2	-	-	-	-	-	-	-	-	-	-	-	-	-	-	-	2	-	1	1	2	-
BoV, CoV-OC43, RV																							
0–4	2 (N; 2; N)	-	2	-	-	2	-	-	-	-	-	-	-	-	-	-	-	-	-	-	-	2	-
BoV, EV, RV																							
0–4	2 (1; 1; N)	-	2	-	-	-	2	-	-	-	-	-	-	-	-	-	-	-	-	-	-	2	-
BoV, RSV																							
0–4	2 (1; 1; N)	-	2	-	-	-	-	-	-	-	-	-	-	-	-	-	-	2	1	-	1	-	-
EV, PiV																							
0–4	2 (1; 1; N)	-	-	-	-	-	2	-	-	-	-	2	-	-	-	1	1	-	-	-	-	-	-
EV, RSV, RV																							
0–4	2 (2; N; N)	-	-	-	-	-	2	-	-	-	-	-	-	-	-	-	-	2	-	-	2	2	-
MPV, RSV																							
0–4	2 (1; 1; N)	-	-	-	-	-	-	-	-	-	2	-	-	-	-	-	-	2	-	-	2	-	-
PiV, RSV																							
0–4 5–14	1 (1; N; N) 1 (1; N; N)	-	-	-	-	-	-	-	-	-	-	2	-	1	-	-	1	2	-	-	2	-	-
PiV, SARS-CoV-2																							
0–4 15–64	1 (1; N; N) 1 (N; 1; N)	-	-	-	-	-	-	-	-	-	-	2	-	-	-	1	1	-	-	-	-	-	2
RSV, SARS-CoV-2																							
0–4 65<	1 (1; N; N) 1 (1; N; N)	-	-	-	-	-	-	-	-	-	-	-	-	-	-	-	-	2	-	-	2	-	2
AdV, BoV, CoV-229E, RV																							
0–4	1 (N; N; 1)	1	1	1	-	-	-	-	-	-	-	-	-	-	-	-	-	-	-	-	-	1	-
AdV, BoV, EV																							
0–4	1 (1; N; N)	1	1	-	-	-	1	-	-	-	-	-	-	-	-	-	-	-	-	-	-	-	-
AdV, BoV, EV, PiV, RV																							
0–4	1 (N; 1; N)	1	1	-	-	-	1	-	-	-	-	1	-	-	-	1	-	-	-	-	-	1	-
AdV, BoV, MPV																							
0–4	1 (N; 1; N)	1	1	-	-	-	-	-	-	-	1	-	-	-	-	-	-	-	-	-	-	-	-
AdV, BoV, MPV, RV																							
0–4	1 (N; 1; N)	1	1	-	-	-	-	-	-	-	1	-	-	-	-	-	-	-	-	-	-	1	-
AdV, BoV, MPV, RV, SARS-CoV-2																							
0–4	1 (N; 1; N)	1	1	-	-	-	-	-	-	-	1	-	-	-	-	-	-	-	-	-	-	1	1
AdV, BoV, PiV, RV																							
0–4	1 (N; N; 1)	1	1	-	-	-	-	-	-	-	-	1	1	-	-	-	-	-	-	-	-	1	-
AdV, BoV, RSV, RV																							
0–4	1 (1; N; N)	1	1	-	-	-	-	-	-	-	-	-	-	-	-	-	-	1	-	-	1	1	-
AdV, BoV, SARS-CoV-2																							
0–4	1 (N; 1; N)	1	1	-	-	-	-	-	-	-	-	-	-	-	-	-	-	-	-	-	-	-	1
AdV, CoV-229E																							
5–14	1 (N; 1; N)	1	-	1	-	-	-	-	-	-	-	-	-	-	-	-	-	-	-	-	-	-	-
AdV, CoV-NL63, RV																							
5–14	1 (N; N; 1)	1	-	-	1	-	-	-	-	-	-	-	-	-	-	-	-	-	-	-	-	1	-
AdV, CoV-OC43, RSV																							
0–4	1 (1; N; N)	1	-	-	-	1	-	-	-	-	-	-	-	-	-	-	-	1	-	-	1	-	-
AdV, CoV-OC43, RV																							
0–4	1 (N; 1; N)	1	-	-	-	1	-	-	-	-	-	-	-	-	-	-	-	-	-	-	-	1	-
AdV, IAV, RV																							
15–64	1 (N; 1; N)	1	-	-	-	-	-	-	1	-	-	-	-	-	-	-	-	-	-	-	-	1	-
AdV, PiV, RSV, RV																							
0–4	1 (1; N; N)	1	-	-	-	-	-	-	-	-	-	1	-	-	-	-	1	1	-	-	1	1	-
AdV, PiV, RV																							
0–4	1 (N; 1; N)	1	-	-	-	-	-	-	-	-	-	1	-	-	-	-	1	-	-	-	-	1	-
AdV, RSV, SARS-CoV-2																							
0–4	1 (N; 1; N)	1	-	-	-	-	-	-	-	-	-	-	-	-	-	-	-	1	-	-	1	-	1
BoV, CoV-NL6, PiV																							
0–4	1 (N; N; 1)	-	1	-	1	-	-	-	-	-	-	1	1	-	-	-	-	-	-	-	-	-	-
BoV, CoV-NL63																							
15–64	1 (N; 1; N)	-	1	-	1	-	-	-	-	-	-	-	-	-	-	-	-	-	-	-	-	-	-
BoV, EV, PiV																							
0–4	1 (1; N; N)	-	1	-	-	-	1	-	-	-	-	1	-	-	-	1	-	-	-	-	-	-	-
BoV, IAV, MPV																							
0–4	1 (N; 1; N)	-	1	-	-	-	-	-	1	-	1	-	-	-	-	-	-	-	-	-	-	-	-
BoV, MPV, PiV																							
0–4	1 (1; N; N)	-	1	-	-	-	-	-	-	-	1	1	-	-	-	-	1	-	-	-	-	-	-
BoV, MPV, RSV																							
0–4	1 (N; 1; N)	-	1	-	-	-	-	-	-	-	1	-	-	-	-	-	-	1	-	-	1	-	-
BoV, MPV, SARS-CoV-2																							
0–4	1 (N; 1; N)	-	1	-	-	-	-	-	-	-	1	-	-	-	-	-	-	-	-	-	-	-	1
BoV, PiV, RSV																							
0–4	1 (N; 1; N)	-	1	-	-	-	-	-	-	-	-	1	-	-	1	-	-	1	-	-	1	-	-
BoV, RSV, RV																							
0–4	1 (1; N; N)	-	1	-	-	-	-	-	-	-	-	-	-	-	-	-	-	1	-	-	1	1	-
BoV, RV, SARS-CoV-2																							
0–4	1 (N; 1; N)	-	1	-	-	-	-	-	-	-	-	-	-	-	-	-	-	-	-	-	-	1	1
BoV, SARS-CoV-2																							
15–64	1 (N; N; 1)	-	1	-	-	-	-	-	-	-	-	-	-	-	-	-	-	-	-	-	-	-	1
CoV-229E, CoV-NL63, CoV-OC43																							
15–64	1 (N; N; 1)	-	-	1	1	1	-	-	-	-	-	-	-	-	-	-	-	-	-	-	-	-	-
CoV-229E, MPV, PiV																							
15–64	1 (N; 1; N)	-	-	1	-	-	-	-	-	-	1	1	1	-	-	-	-	-	-	-	-	-	-
CoV-229E, RV																							
5–14	1 (N; 1; N)	-	-	1	-	-	-	-	-	-	-	-	-	-	-	-	-	-	-	-	-	1	-
CoV-NL63, EV/RV																							
15–64	1 (N; 1; N)	-	-	-	1	-	-	1	-	-	-	-	-	-	-	-	-	-	-	-	-	-	-
CoV-OC4, IAV, SARS-CoV-2																							
5–14	1 (N; 1; N)	-	-	-	-	1	-	-	1	-	-	-	-	-	-	-	-	-	-	-	-	-	1
CoV-OC4, PiV																							
15–64	1 (N; N; 1)	-	-	-	-	1	-	-	-	-	-	1	1	-	-	-	-	-	-	-	-	-	-
CoV-OC43, RV, SARS-CoV-2																							
5–14	1 (N; 1; N)	-	-	-	-	1	-	-	-	-	-	-	-	-	-	-	-	-	-	-	-	1	1
EV, PiV, RV																							
0–4	1 (1; N; N)	-	-	-	-	-	1	-	-	-	-	1	-	1	-	-	-	-	-	-	-	1	-
EV, RV, SARS-CoV-2																							
0–4	1 (1; N; N)	-	-	-	-	-	1	-	-	-	-	-	-	-	-	-	-	-	-	-	-	1	1
EV/RV, IAV, SARS-CoV-2																							
65<	1 (N; 1; N)	-	-	-	-	-	-	1	1	-	-	-	-	-	-	-	-	-	-	-	-	-	1
EV/RV, PiV																							
15–64	1 (1; N; N)	-	-	-	-	-	-	1	-	-	-	1	-	1	-	-	-	-	-	-	-	-	-
EV/RV, SARS-CoV-2																							
15–64	1 (1; N; N)	-	-	-	-	-	-	1	-	-	-	-	-	-	-	-	-	-	-	-	-	-	1
IAV, RSV, RV																							
0–4	1 (N; 1; N)	-	-	-	-	-	-	-	1	-	-	-	-	-	-	-	-	1	-	-	1	1	-
IAV, RV, SARS-CoV-2																							
5–14	1 (N; 1; N)	-	-	-	-	-	-	-	1	-	-	-	-	-	-	-	-	-	-	-	-	1	1
IVB, MPV																							
0–4	1 (N; 1; N)	-	-	-	-	-	-	-	-	1	1	-	-	-	-	-	-	-	-	-	-	-	-
MPV, PiV																							
0–4	1 (1; N; N)	-	-	-	-	-	-	-	-	-	1	1	-	-	-	-	1	-	-	-	-	-	-
MPV, SARS-CoV-2																							
0–4	1 (N; 1; N)	-	-	-	-	-	-	-	-	-	1	-	-	-	-	-	-	-	-	-	-	-	1
