# Supplementary figures and images for: Circulation and Codetections of Influenza Virus, SARS-CoV-2, Respiratory Syncytial Virus, Rhinovirus, Adenovirus, Bocavirus, and Other Respiratory Viruses During 2022–2023 Season in Latvia
Source: Viruses. 2024 Oct 22;16(11):1650. doi: 10.3390/v16111650 (PMC11598885; doi:10.3390/v16111650)

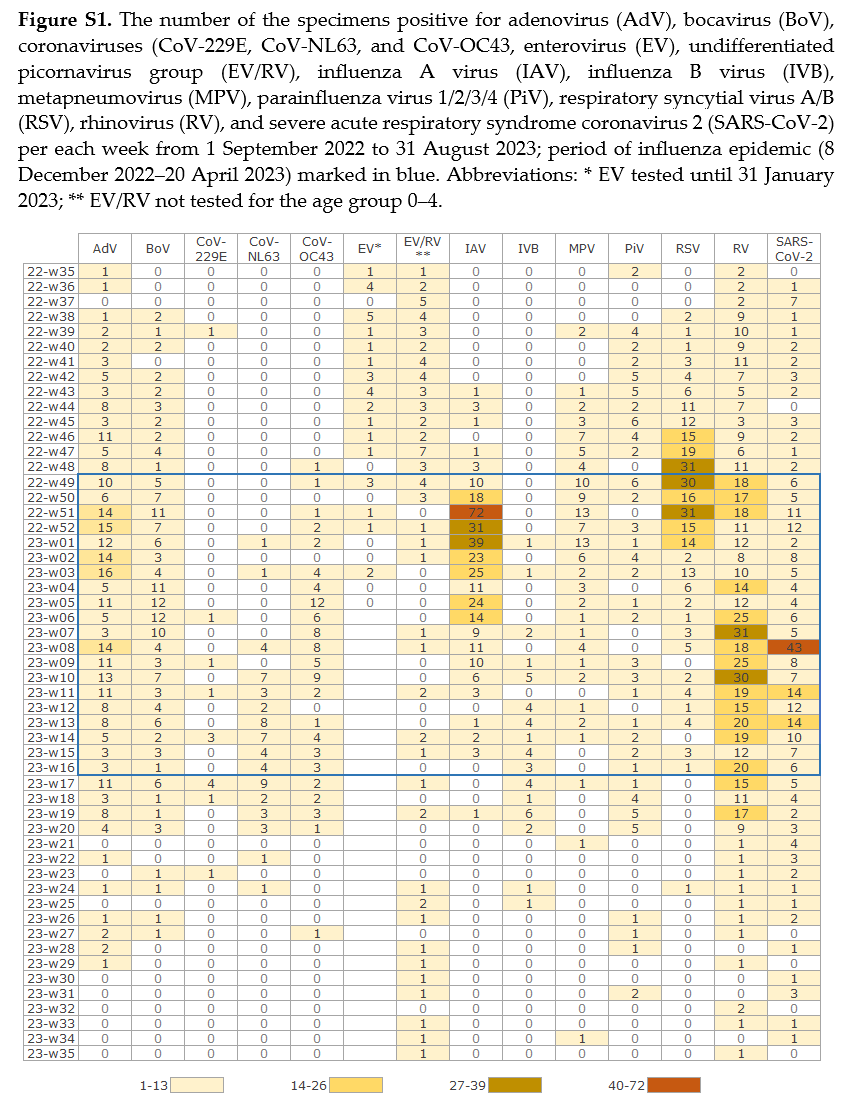

Supplement: Supplementary file 1 [file viruses-16-01650-s001.zip › viruses-3232541-supplementary.png]
